# Supplementary material for: Reactant friendly hydrogen evolution interface based on di-anionic MoS2 surface
Source: Nat Commun. 2020 Feb 28;11:1116. doi: 10.1038/s41467-020-14980-z (PMC7048837; doi:10.1038/s41467-020-14980-z)
Supplement: Supplementary file 1 — Supporting Information [file 41467_2020_14980_MOESM1_ESM.pdf]

Supplementary Information for

Reactant friendly hydrogen evolution interface based on di-anionic MoS_2 surface

Luo *et al.*

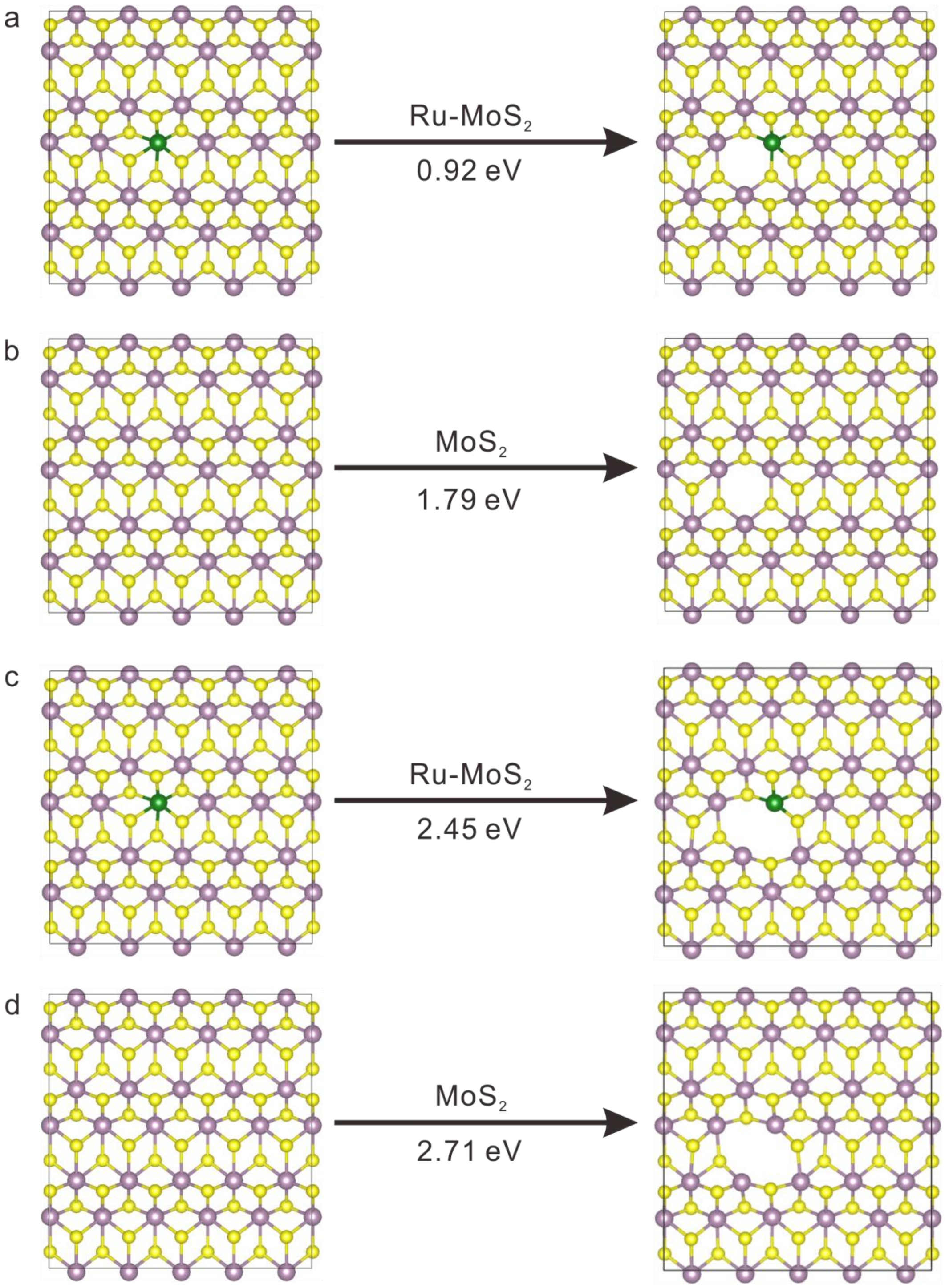

Supplementary Figure 1| Formation energy of S-vacancy. a Formation of single sulfur defects sites (1SVs) in Ru-MoS_2. **b** Formation of single sulfur defects sites (1SVs) in MoS_2. **c** Formation of double sulfur defects sites (2SVs) in Ru-MoS_2. **d** Formation of double sulfur defects sites (2SVs) in MoS_2.

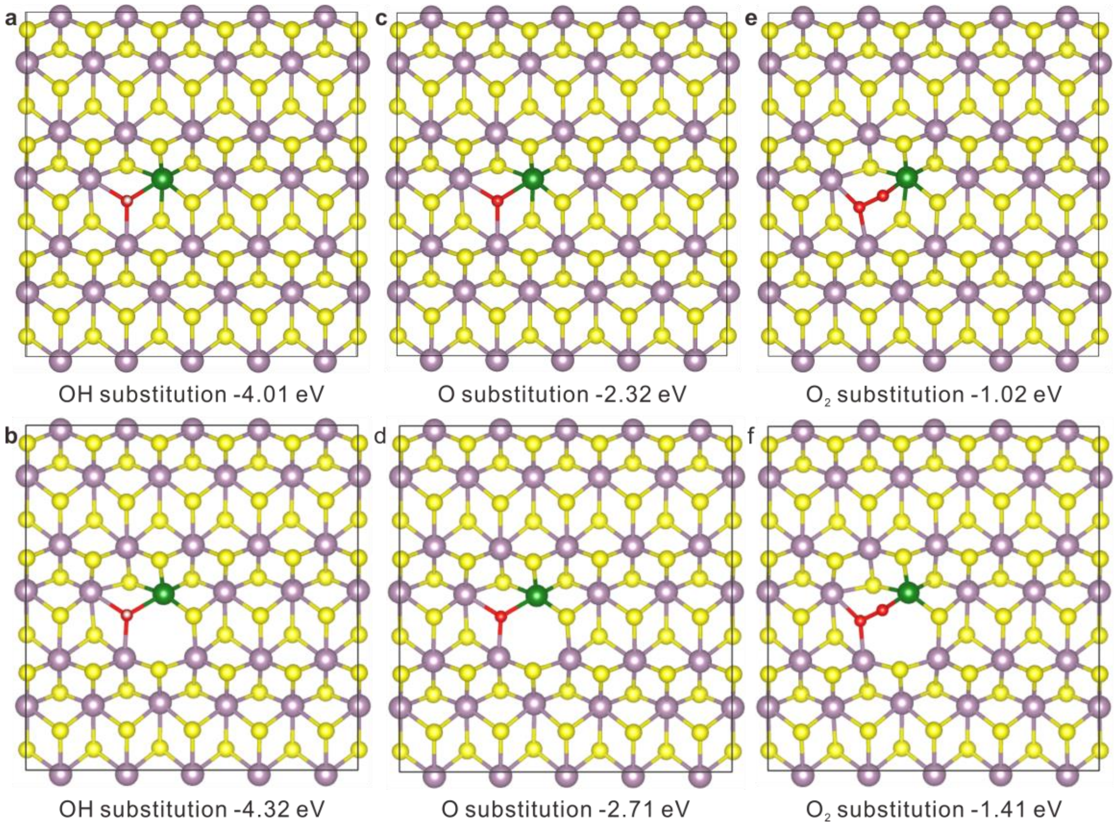

Supplementary Figure 2| DFT calculation. a-b The formation energies of OH occupying the Ru bonded defects sites in Ru-MoS_2; **c-d** The formation energies of O occupying the Ru bonded defects sites in Ru-MoS_2; **e-f** The formation energies of O_2 group occupying the Ru bonded defects sites in Ru-MoS_2.

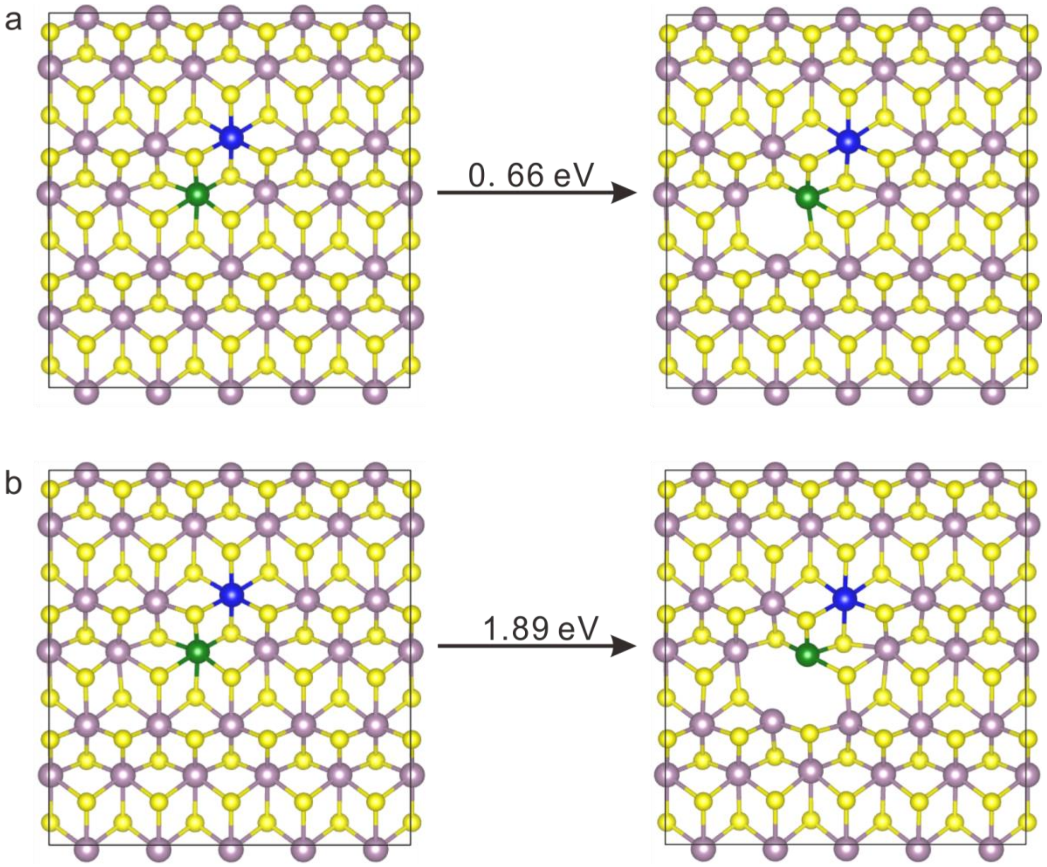

Supplementary Figure 3| Formation energy of S-vacancy. a Formation of single sulfur defects sites (1SVs) in Pd,Ru-MoS_2. **b** Formation of double sulfur defects sites (2SVs) in Pd,Ru-MoS_2.

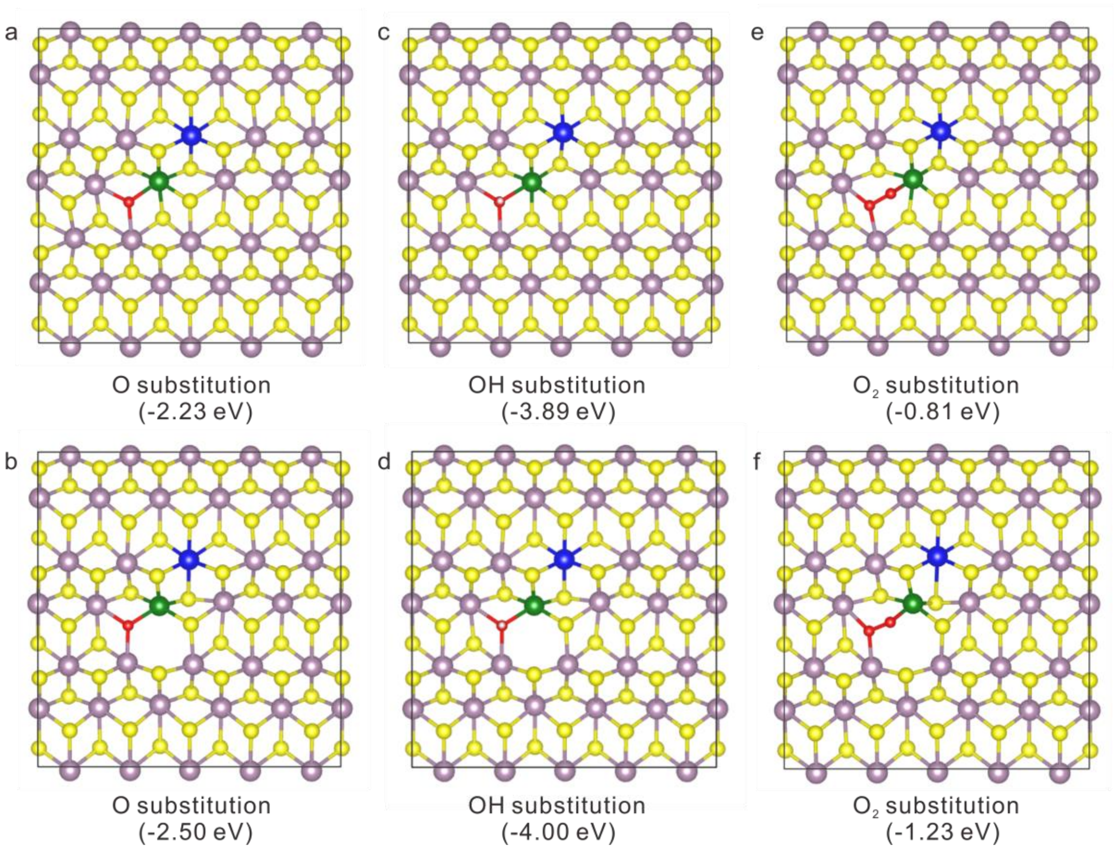

Supplementary Figure 4| DFT calculation. a-b The formation energies of O occupying the Ru bonded defects sites in Pd,Ru-MoS_2. **c-d** The formation energies of OH occupying the Ru bonded defects sites Pd,Ru-MoS_2. **e-f** The formation energies of O_2 group occupying the Ru bonded defects sites Pd,Ru-MoS_2.

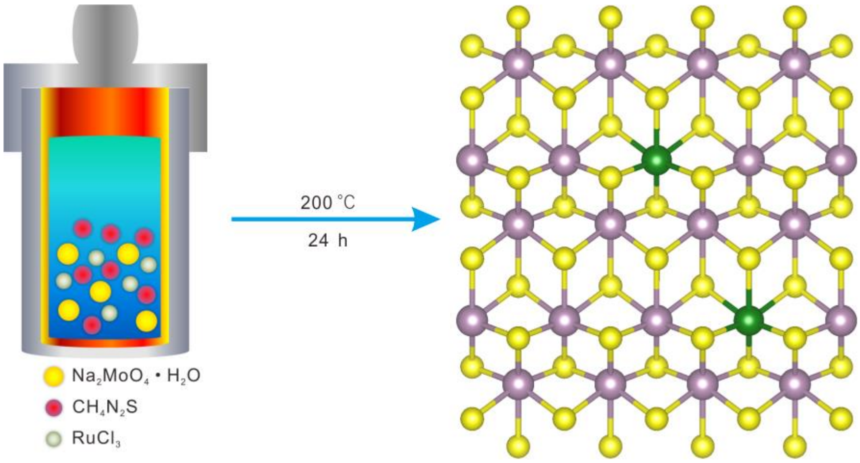

Supplementary Figure 5|Scheme. Schematic illustration of the preparation of Ru-doped molybdenum disulfide (Ru-MoS_2).

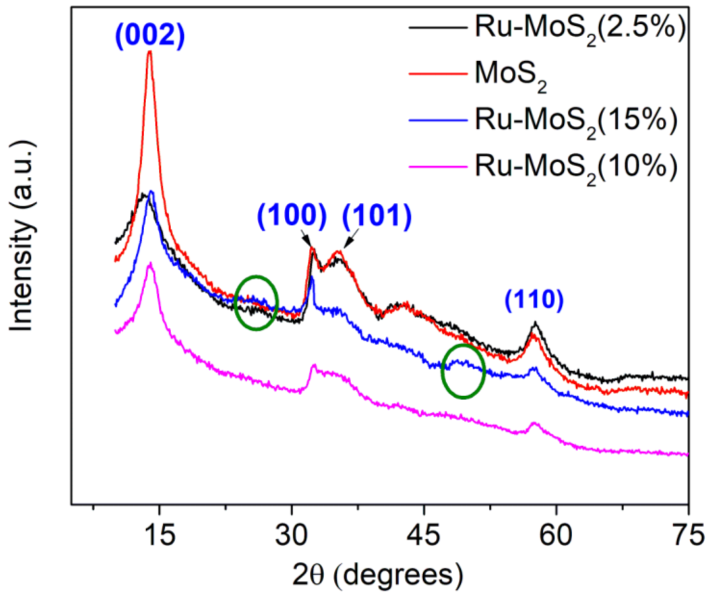

Supplementary Figure 6| XRD patterns. XRD patterns of MoS_2, Ru-MoS_2 (2.5%), Ru-MoS_2 (10%) and Ru-MoS_2 (15%) samples. Four main diffraction peaks around 14 ℃, 33 ℃, 37 ℃ and 58 ℃ are noticed, corresponding to (002), (100), (101) and (110) planes of MoS_2 crystal, respectively.

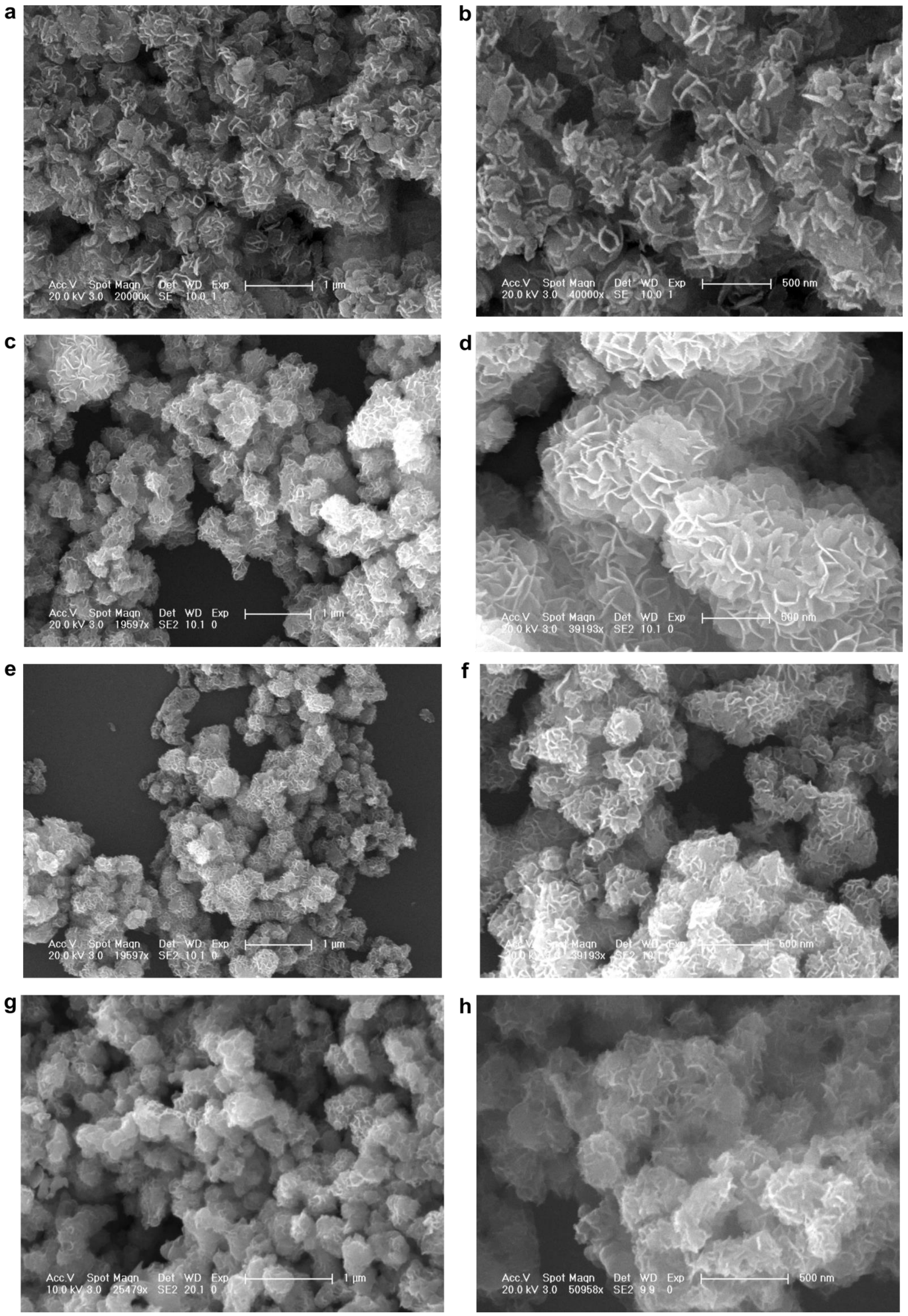

Supplementary Figure 7| SEM patterns. a-b SEM of MoS_2. **c-d**. SEM of Ru-MoS_2 (2.5%). **e-f**. SEM of Ru-MoS_2(10%). **g-h** SEM of Ru-MoS_2(15%).

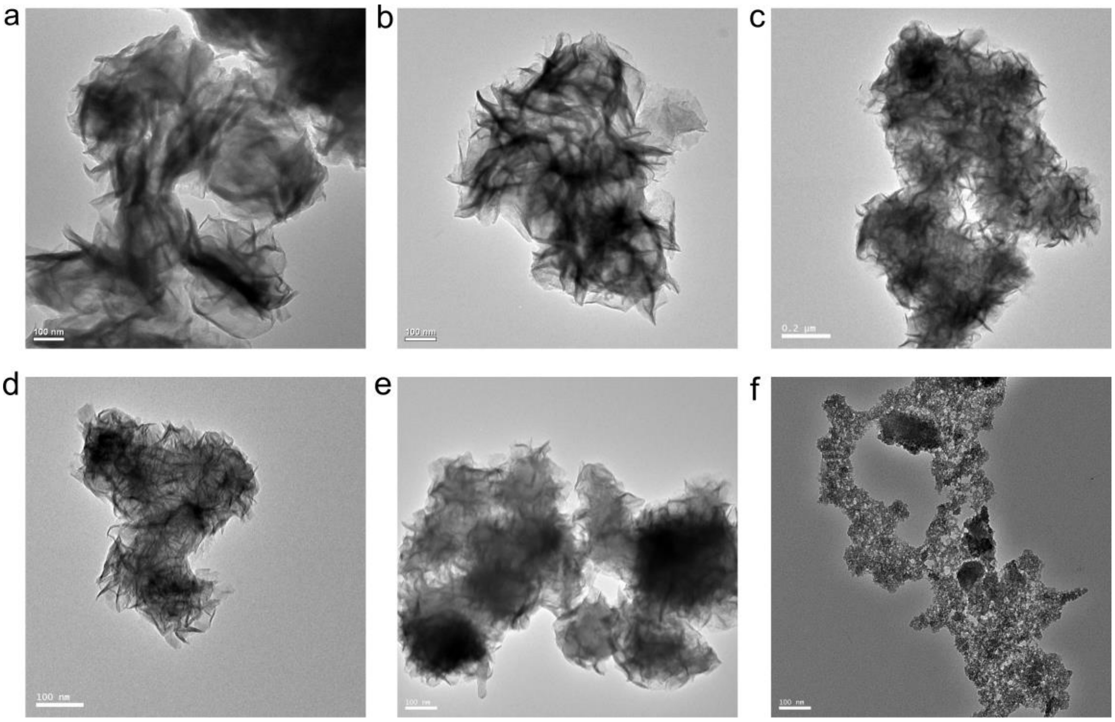

Supplementary Figure 8| TEM patterns. a TEM of MoS_2. **b** TEM of Ru-MoS_2(2.5%). **c** TEM of Ru-MoS_2(5%). **d** TEM of Ru-MoS_2(10%). **e** TEM of Ru-MoS_2(15%). **f** TEM of RuSx.

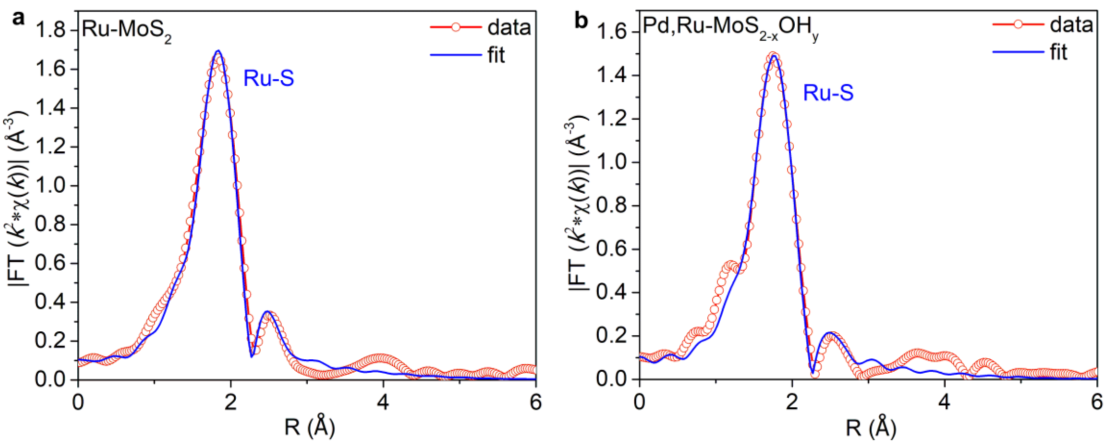

Supplementary Figure 9| EXAFS spectra. a Fourier transforms of k^2-weighted Ru K-edge EXAFS spectra of Ru-MoS_2. **b** Fourier transforms of k^2-weighted Ru K-edge EXAFS spectra of Pd,Ru-$MoS_{2-x}OH_y$. The blue solid line are experimental results, and the red dotted line are best-fit curves for R = 1.0–3.0 Å, using corresponding $k^2\ \chi(k)$ functions in k = 3.0–11.3.0 $Å^{-1}$.

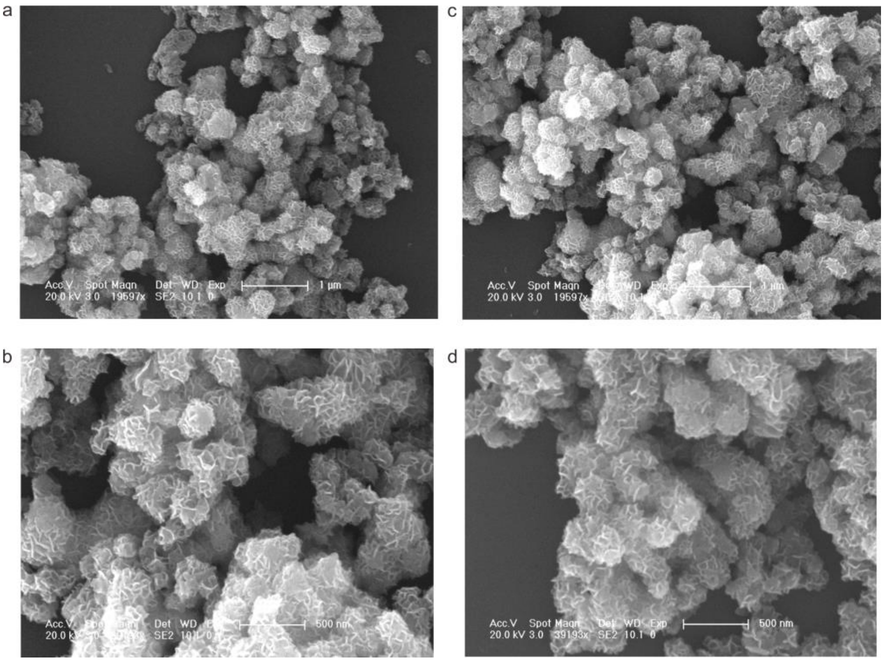

Supplementary Figure 10| SEM patterns. a-b SEM of Ru-MoS_2, the corresponding scale bars are 1 um. **c-d** SEM of Pd,Ru-$MoS_{2-x}OH_y$, the corresponding scale bars are 500 nm.

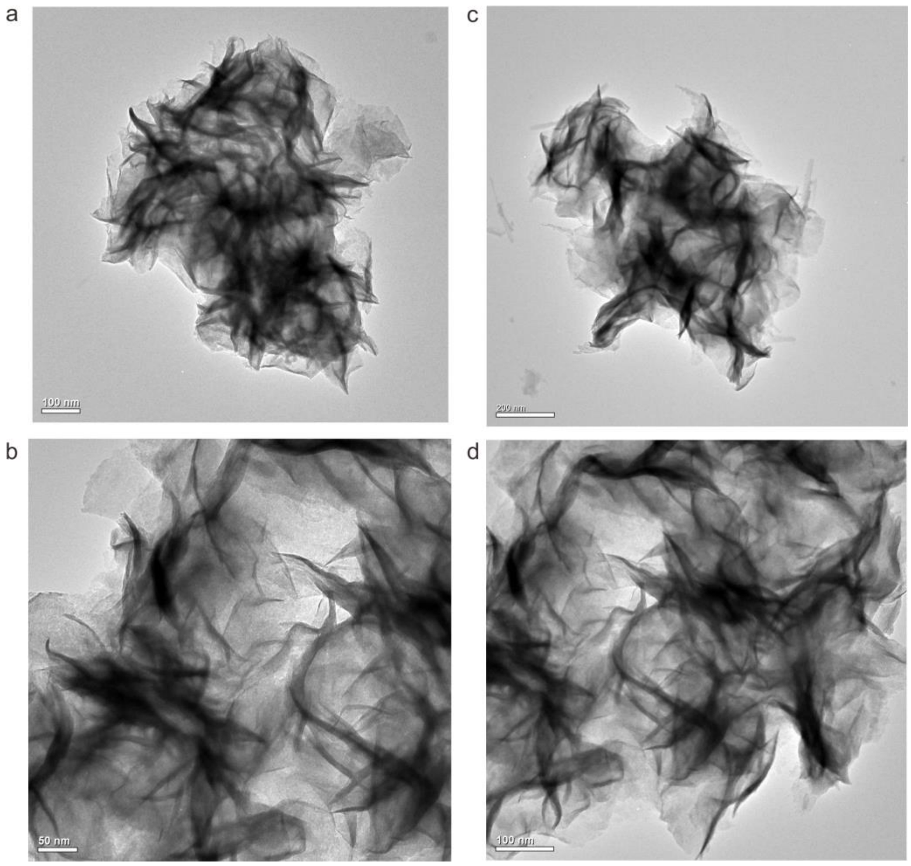

Supplementary Figure 11| TEM patterns. a-b TEM of Ru-MoS_2**. c-d** TEM of Pd,Ru-$MoS_{2-x}OH_y$.

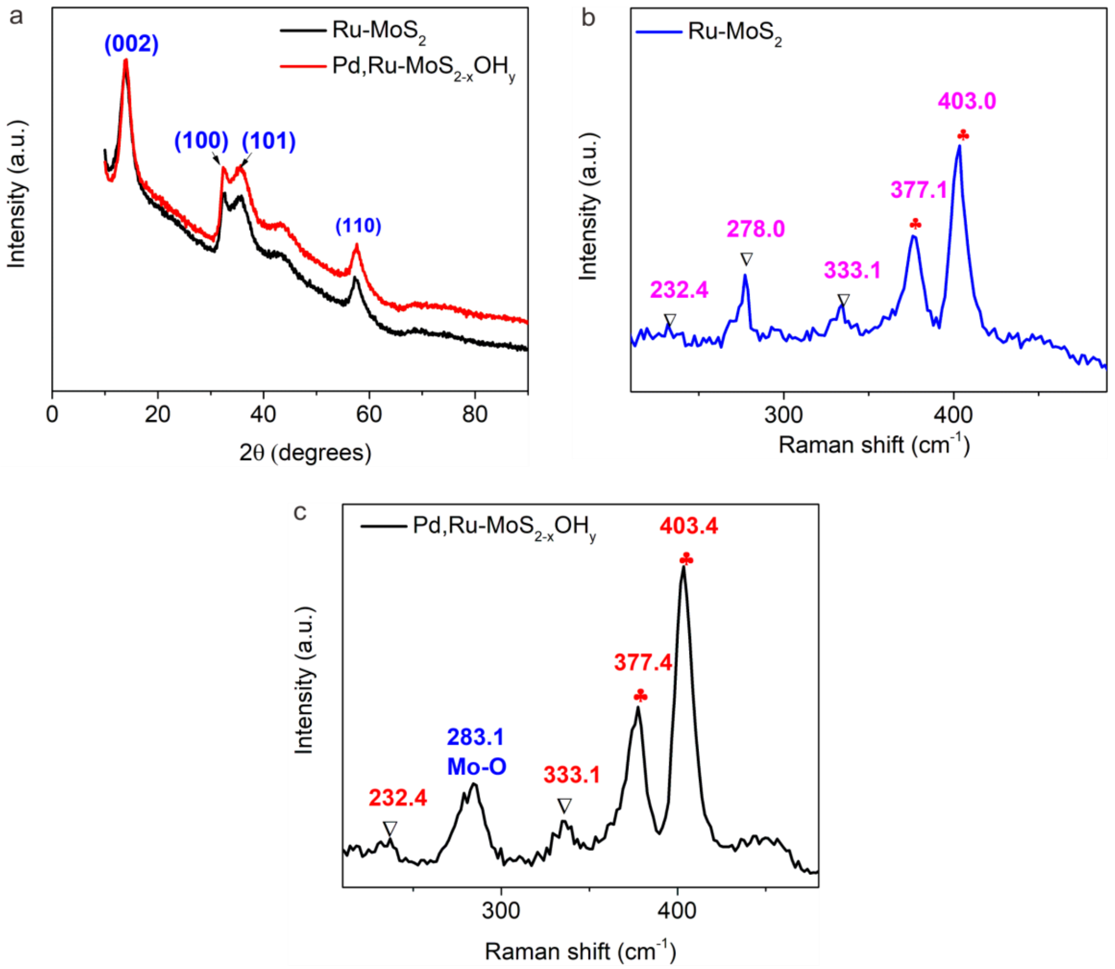

Supplementary Figure 12|XRD pattern and Ramam patterns for samples. a XRD patterns of Ru-MoS_2 and Pd,Ru-$MoS_{2-x}OH_y$. Four main diffraction peaks around 14 ℃, 33 ℃, 37 ℃ and 58 ℃ are noticed, corresponding to (002), (100), (101) and (110) planes of MoS_2 crystal, respectively. **b-c** Raman spectra of Ru-MoS_2 and Pd,Ru-$MoS_{2-x}OH_y$ samples**.** (The two distinct peaks at 378 cm^{-1} and 404 cm^{-1}, corresponding to the 2H phase vibrational configurations of the in-plane Mo-S phonon mode (E_{2g}) and the out-of-plane Mo-S mode (A_{1g}); the three extra peaks at ~232, 278 and 333 cm^{-1}, attributable to J_2, E_{1g}, and J_3 peaks of the 1T phase MoS_2 phonon modes, respectively)

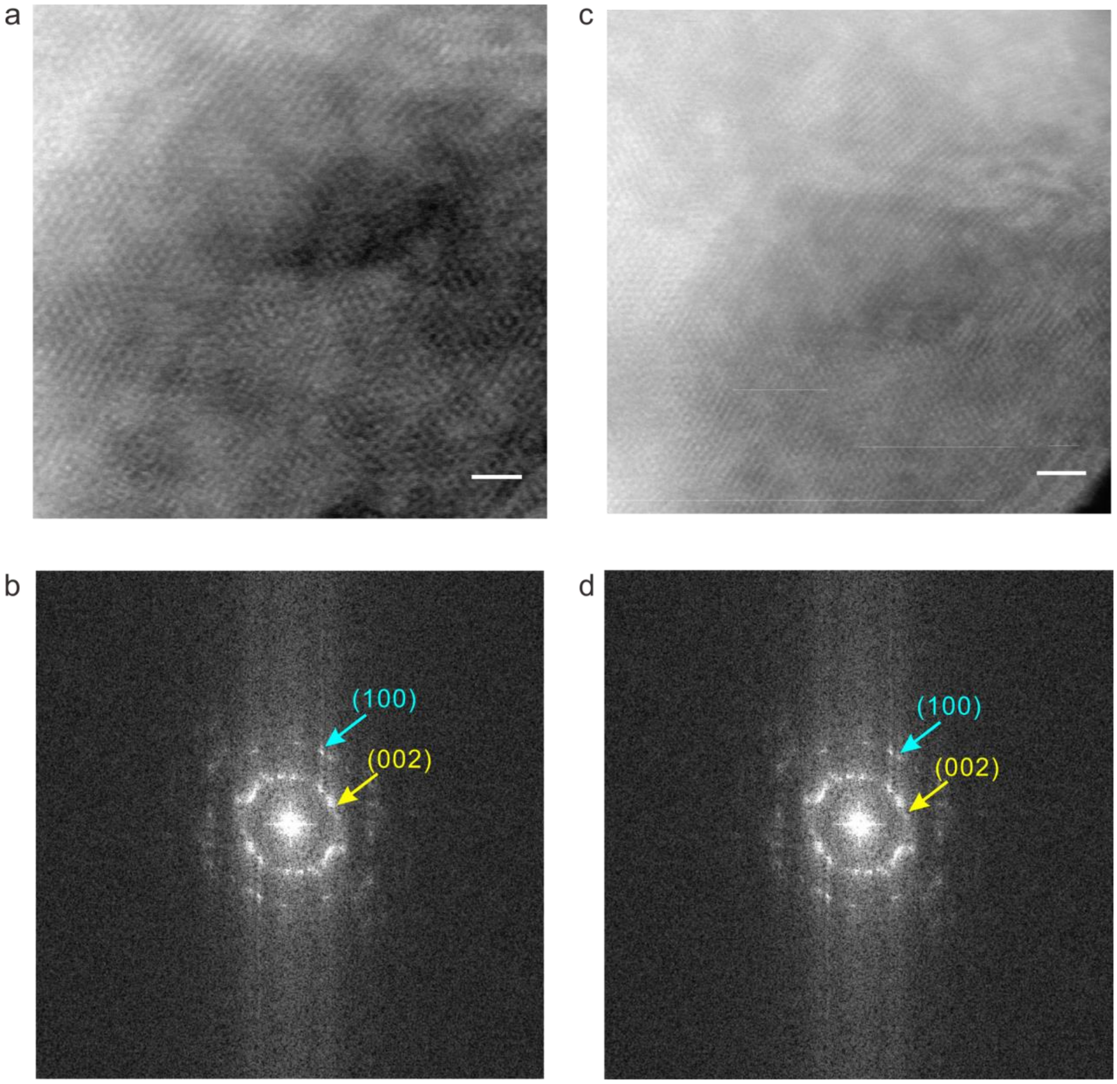

Supplementary Figure 13|HAADF-STEM patterns. a The sub-angstron resolution aberration-corrected HAADF-STEM images of Ru-MoS_2. **b** The SAED pattern of Ru-MoS_2, with the rings corresponding to the expected lattice spacings of MoS_2, representing the (002), (100) reflections for MoS_2, respectively. **c** The sub-angstron resolution aberration-corrected HAADF-STEM images of Pd,Ru-$MoS_{2-x}OH_y$. **d** The SAED pattern of Pd,Ru-$MoS_{2-x}OH_y$, with the rings corresponding to the expected lattice spacings of MoS_2, representing the (002), (100) reflections for MoS_2, respectively.

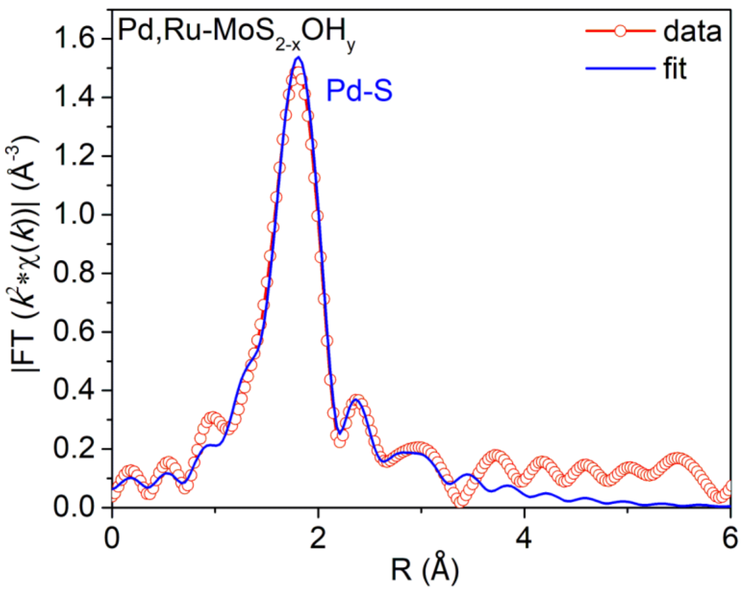

Supplementary Figure 14| EXAFS spectra. Fourier transforms of k^2-weighted Pd K-edge EXAFS spectra of Pd,Ru-$MoS_{2-x}OH_y$. The black solid line are experimental results, and the red dotted line are best-fit curves for R = 1.0-3.3 Å, using corresponding k^2 $\chi(k)$ functions in k = 2.8–11.4 $Å^{-1}$.

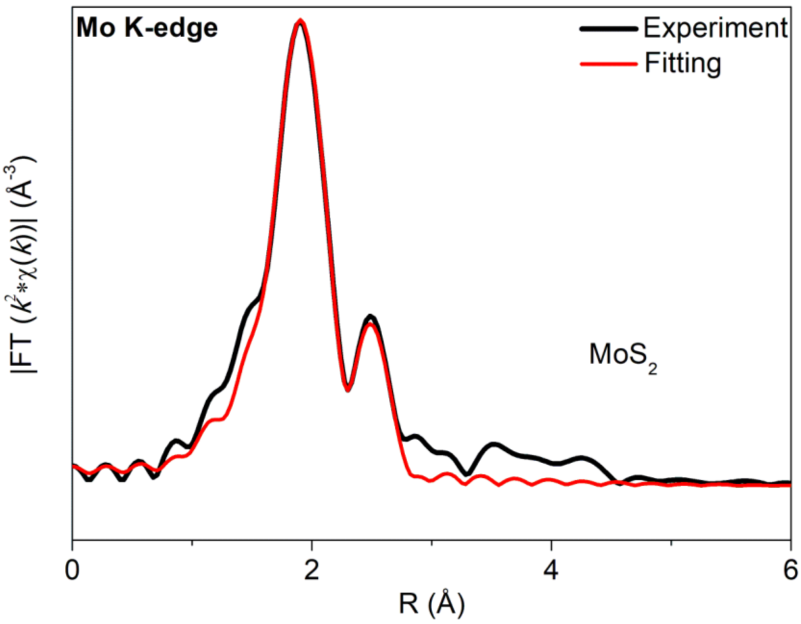

Supplementary Figure 15| EXAFS spectra. Fourier transforms of k^2-weighted Mo K-edge EXAFS spectra of MoS_2. The black line is experimental results, and the red line is best-fit curves for R = 1.1–3.3 Å, using corresponding k^2 $\chi(k)$ functions in k = 3.0–12.0 $Å^{-1}$.

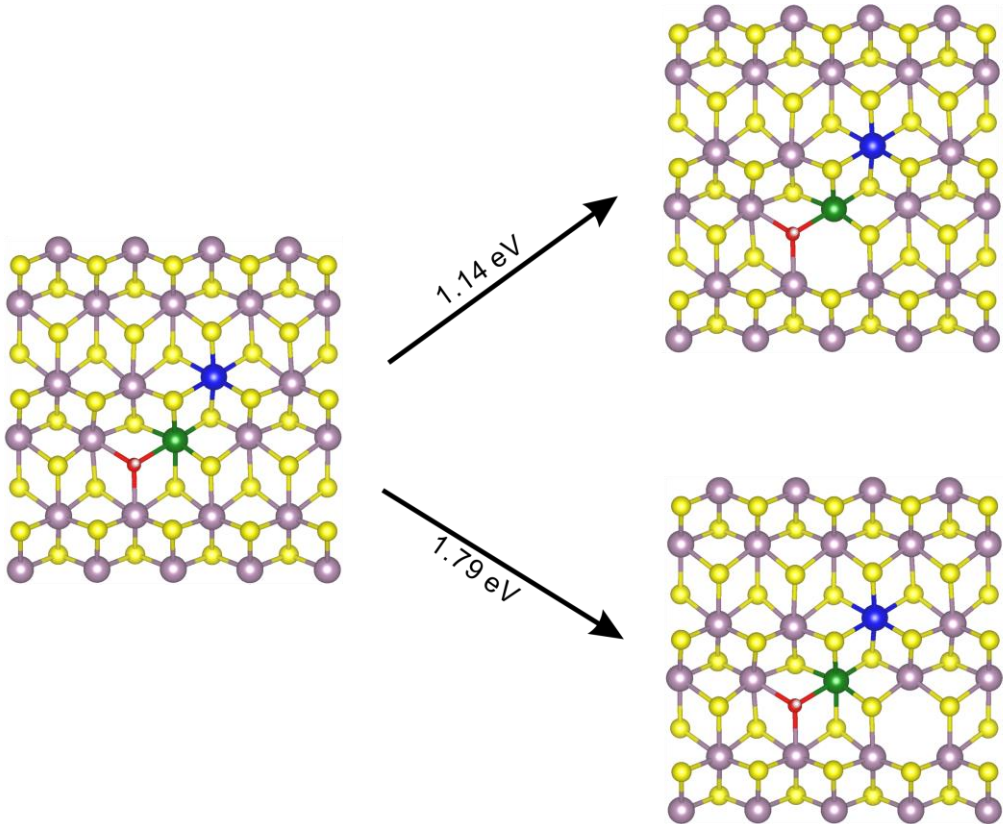

Supplementary Figure 16|DFT calculations. Formation energy of S-vacancy.

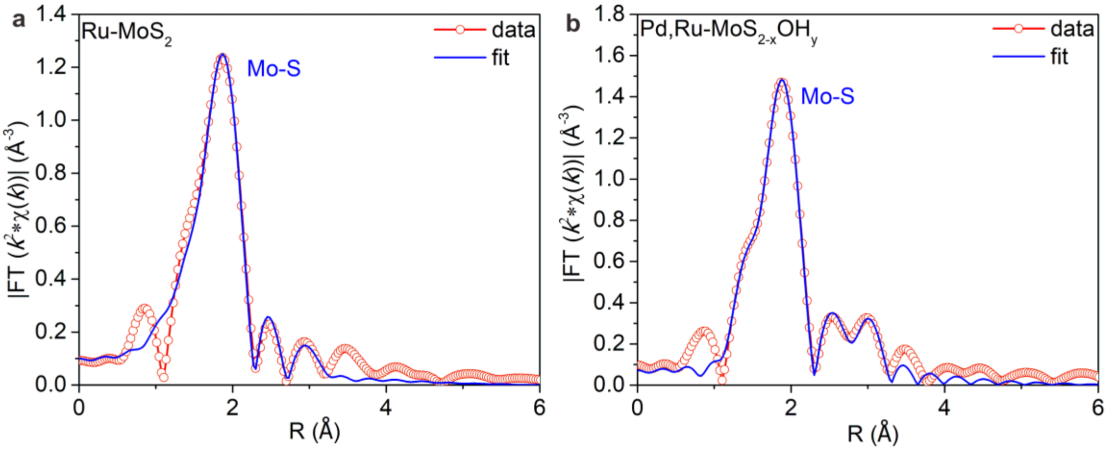

Supplementary Figure 17|EXAFS spectra. a Fourier transforms of k^2-weighted Mo K-edge EXAFS spectra of Ru-MoS_2. **b** Fourier transforms of k^2-weighted Mo K-edge EXAFS spectra of Pd,Ru-$MoS_{2-x}OH_y$. The blue solid line are experimental results, and the red dotted line are best-fit curves for R = 1.1–3.3 Å, using corresponding k^2 $\chi(k)$ functions in k = 3.0–12.0 $Å^{-1}$.

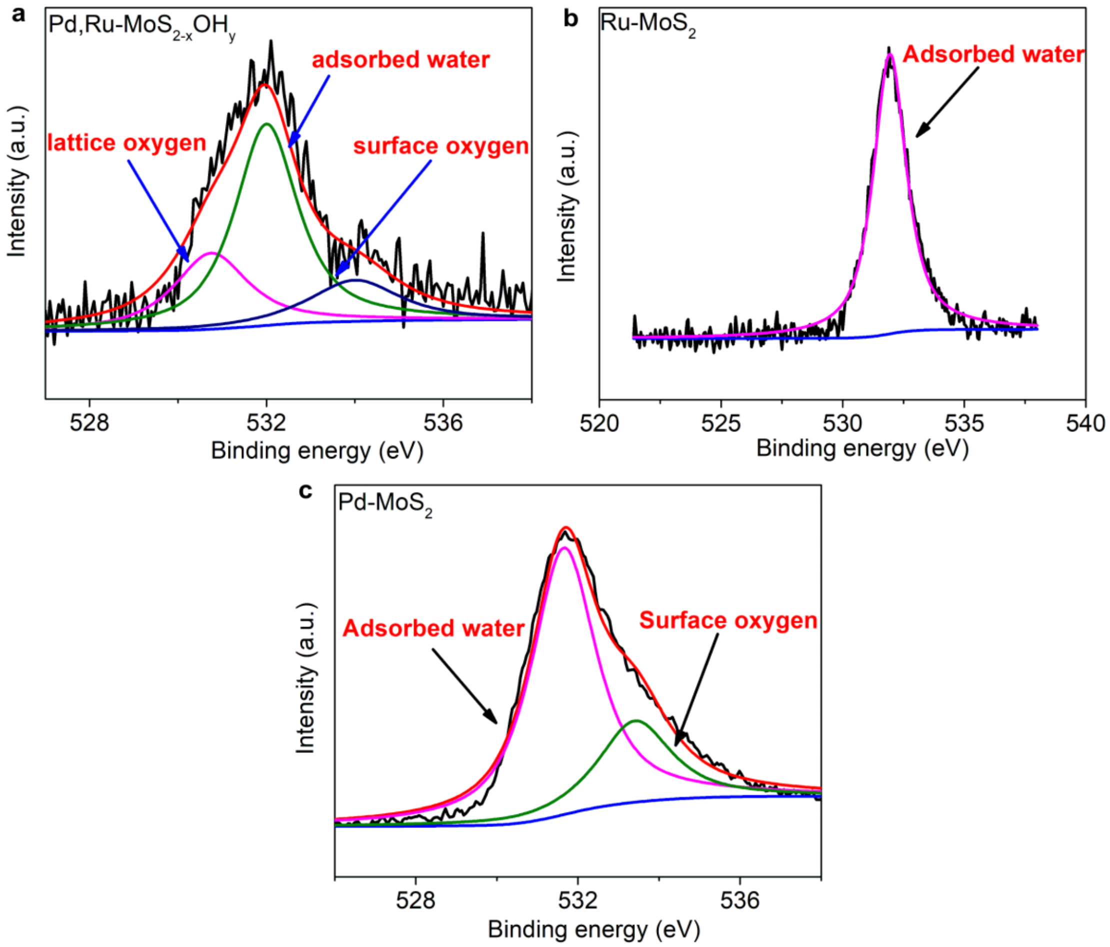

Supplementary Figure 18|XPS spectra. **a** High-resolution XPS results (O *1s* region) of the Pd,Ru-$MoS_{2-x}OH_y$. **b** High-resolution XPS results (O *1s* region) of the Ru-MoS_2. **c** High-resolution XPS results (O *1s* region) of the Pd-MoS_2.

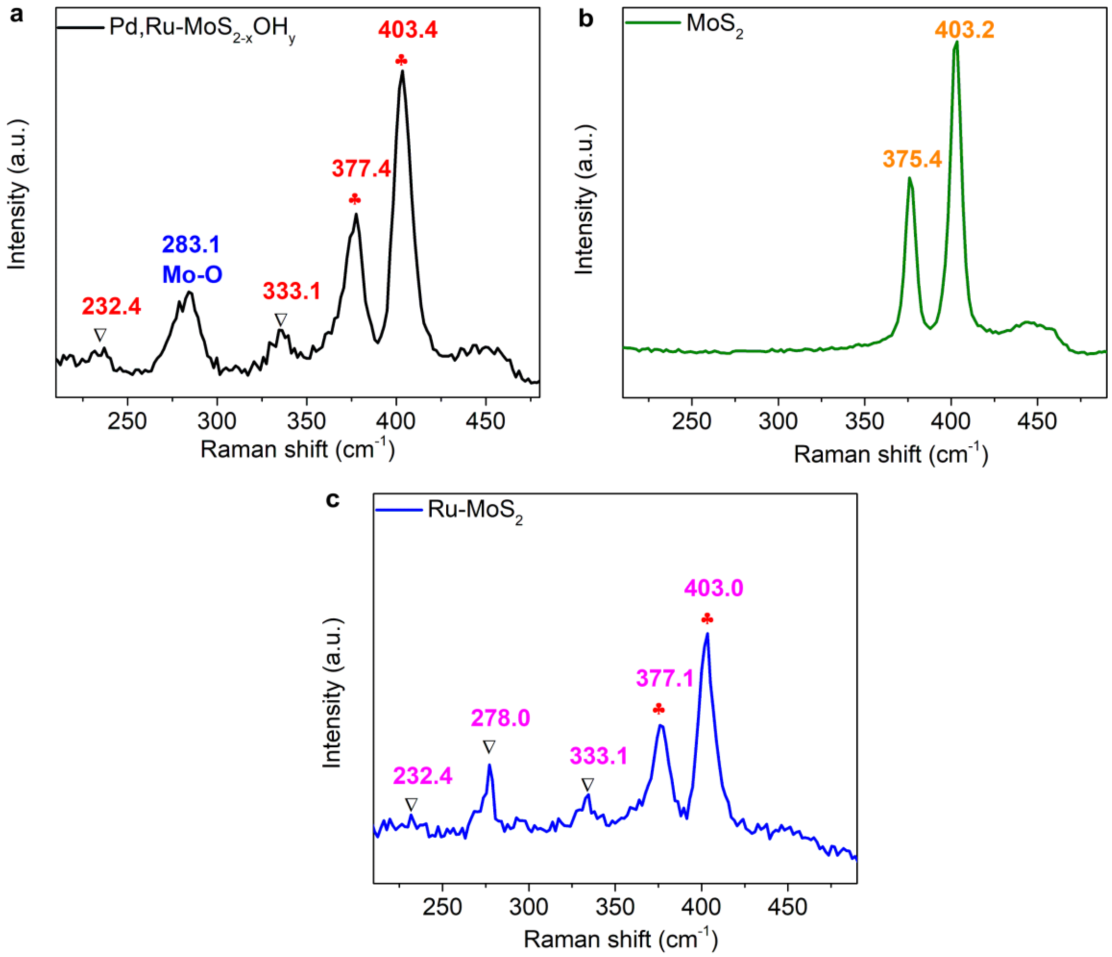

Supplementary Figure 19| Raman spectra. a Raman spectra of Pd,Ru-$MoS_{2-x}OH_y$. **b** Raman spectra of MoS_2. **c** Raman spectra of Ru-MoS_2**.** (The two distinct peaks at 378 cm^{-1} and 404 cm^{-1}, corresponding to the 2H phase vibrational configurations of the in-plane Mo-S phonon mode (E_{2g}) and the out-of-plane Mo-S mode (A_{1g}); the three extra peaks at ~232, 278 and 333 cm^{-1}, attributable to J_2, E_{1g}, and J_3 peaks of the 1T phase MoS_2 phonon modes, respectively)

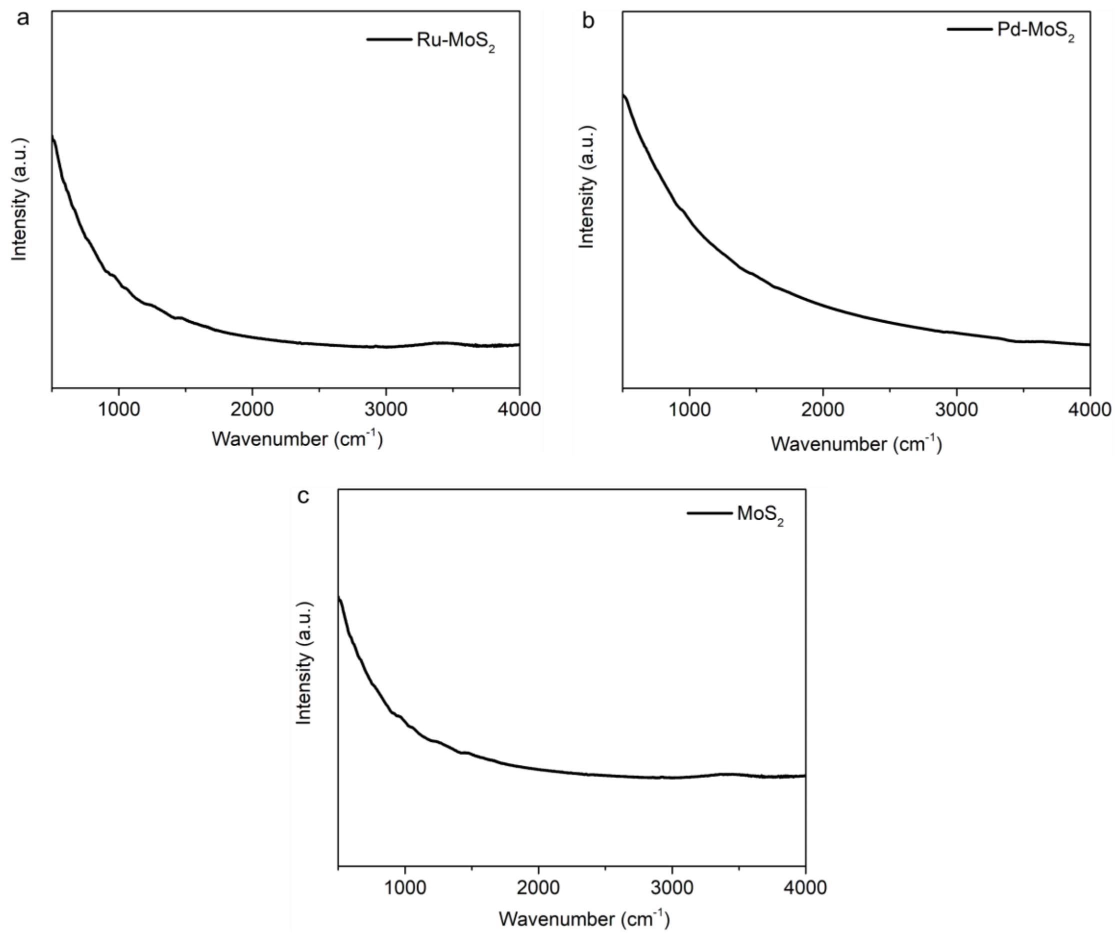

Supplementary Figure 20| FTIR spectra. a FTIR spectra of Ru-MoS_2. **b** FTIR spectra of Pd-MoS_2. **c** FTIR spectra of MoS_2. (FTIR spectra were used to identify the specific configuration of O in the sample. The samples were pretreated under vacuum condition to eliminate water in the sample)

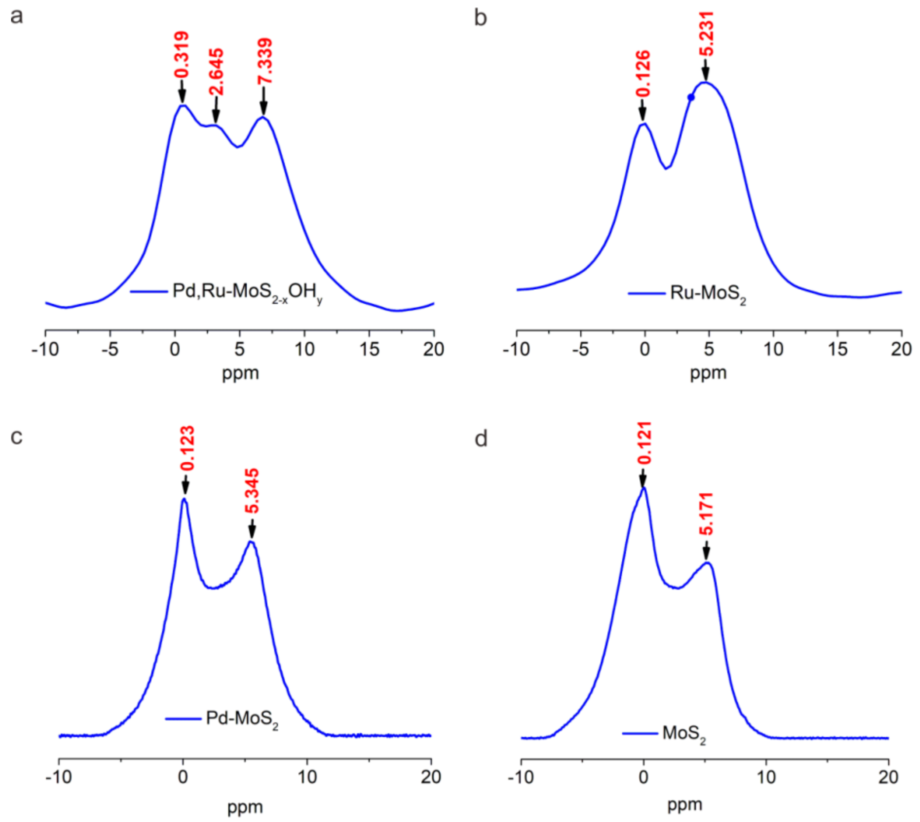

Supplementary Figure 21| proton solid-state nuclear magnetic resonance (^{1}H SS-NMR) spectrum.

a ^{1}H SS-NMR spectra of Pd,Ru-$MoS_{2-x}OH_y$. **b** ^{1}H SS-NMR spectra of Ru-MoS_2. **c** ^{1}H SS-NMR spectra of Pd-MoS_2. **d** ^{1}H SS-NMR spectra of MoS_2.

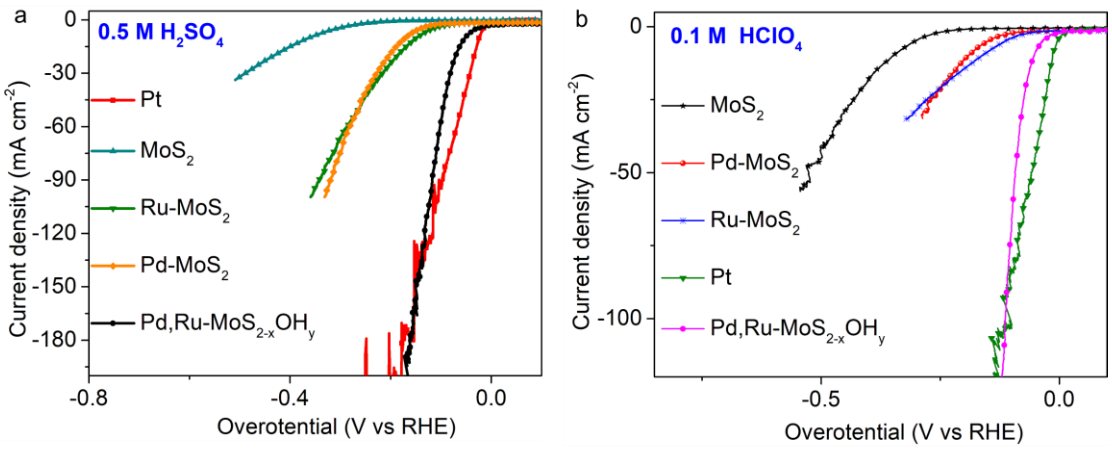

Supplementary Figure 22| LSV polarization curves. **a** LSV polarization curves of Pt, MoS_2, Ru-MoS_2, Pd-MoS_2 and Pd,Ru-$MoS_{2-x}OH_y$ in 0.5 M H_2SO_4 (with *iR* correction). **b** LSV polarization curves of Pt, MoS_2, Ru-MoS_2, Pd-MoS_2 and Pd,Ru-$MoS_{2-x}OH_y$ in 0.1 M $HClO_4$ (with *iR* correction).

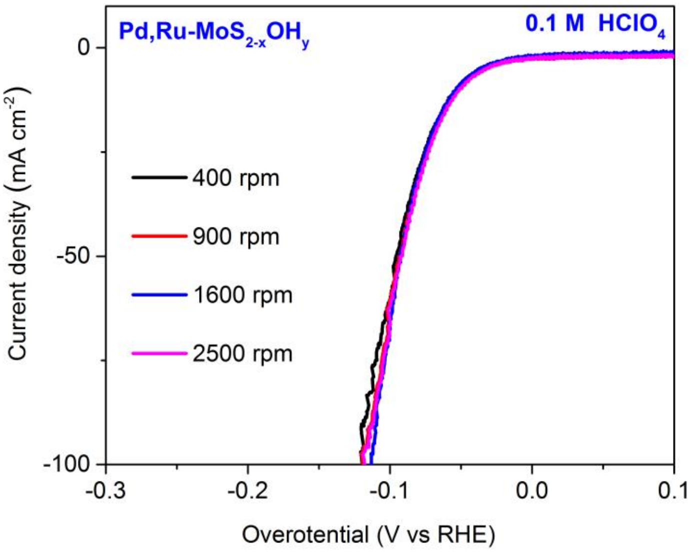

Supplementary Figure 23| LSV polarization curves. *iR*-corrected HER polarization curves at different rotation speeds (400 rpm to 2500 rpm) on Pd,Ru-$Mo_{2-x}OH_y$ catalyst in H_2–saturated 0.1 M $HClO_4$ at a scanning rate of 10 mV/s. (The HER measurement results show a minimum influence of H_2 mass transportation on the final catalytic behavior. Therefore, the catalytic performance of the catalyst is kinetic controlled on our catalysts)

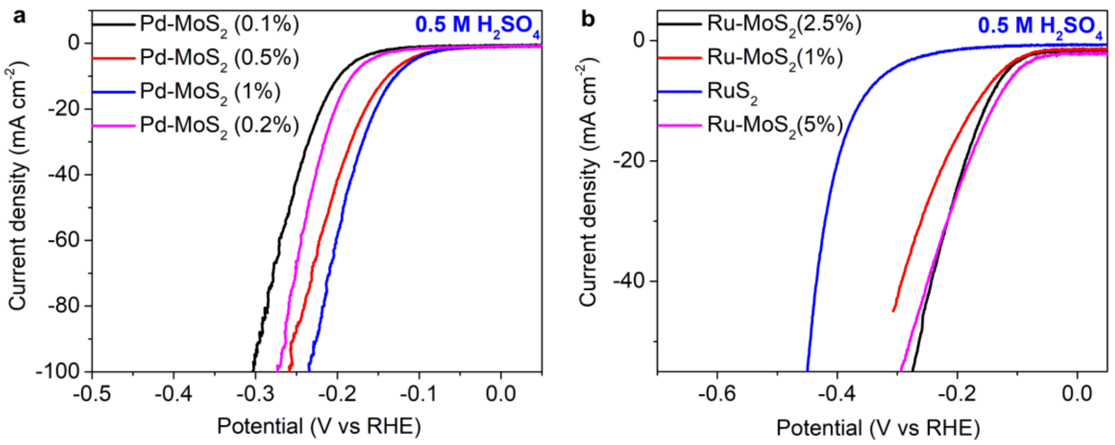

Supplementary Figure 24| HER performance. a LSV polarization curves of Pd-MoS_2 samples with different Pd loading. **b** LSV polarization curves of Ru-MoS_2 samples with different Ru loading and RuS_2.

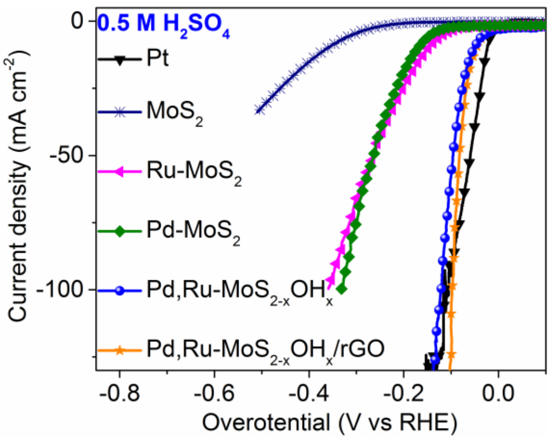

Supplementary Figure 25| HER performance. LSV polarization curves of Pt, MoS_2, Ru-MoS_2, Pd-MoS_2, Pd,Ru-$MoS_{2-x}OH_y$ and Pd,Ru-$MoS_{2-x}OH_y$/rGO in 0.5 M H_2SO_4. (with *iR* correction).

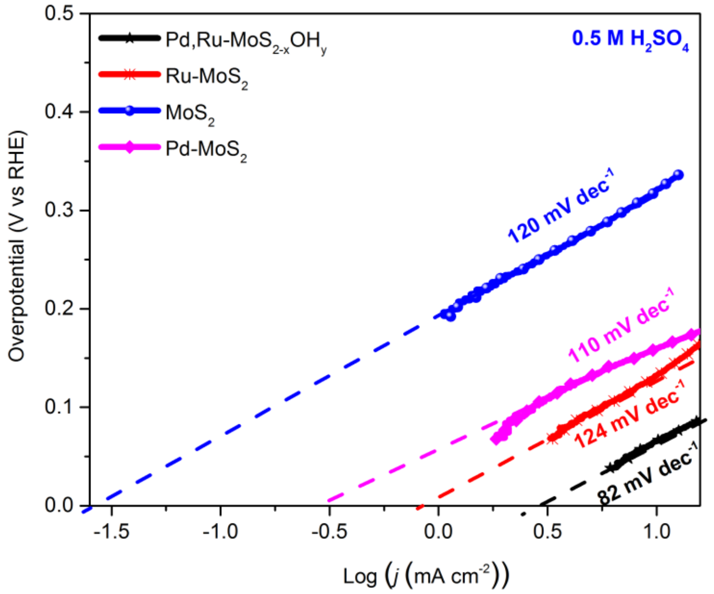

Supplementary Figure 26| Exchange current density. Exchange current density for the MoS_2, Pd–MoS_2, Ru–MoS_2 and Pd,Ru-$MoS_{2-x}OH_y$ samples in 0.5 M H_2SO_4, derived from the Tafel plots, as indicated by the dashed lines.

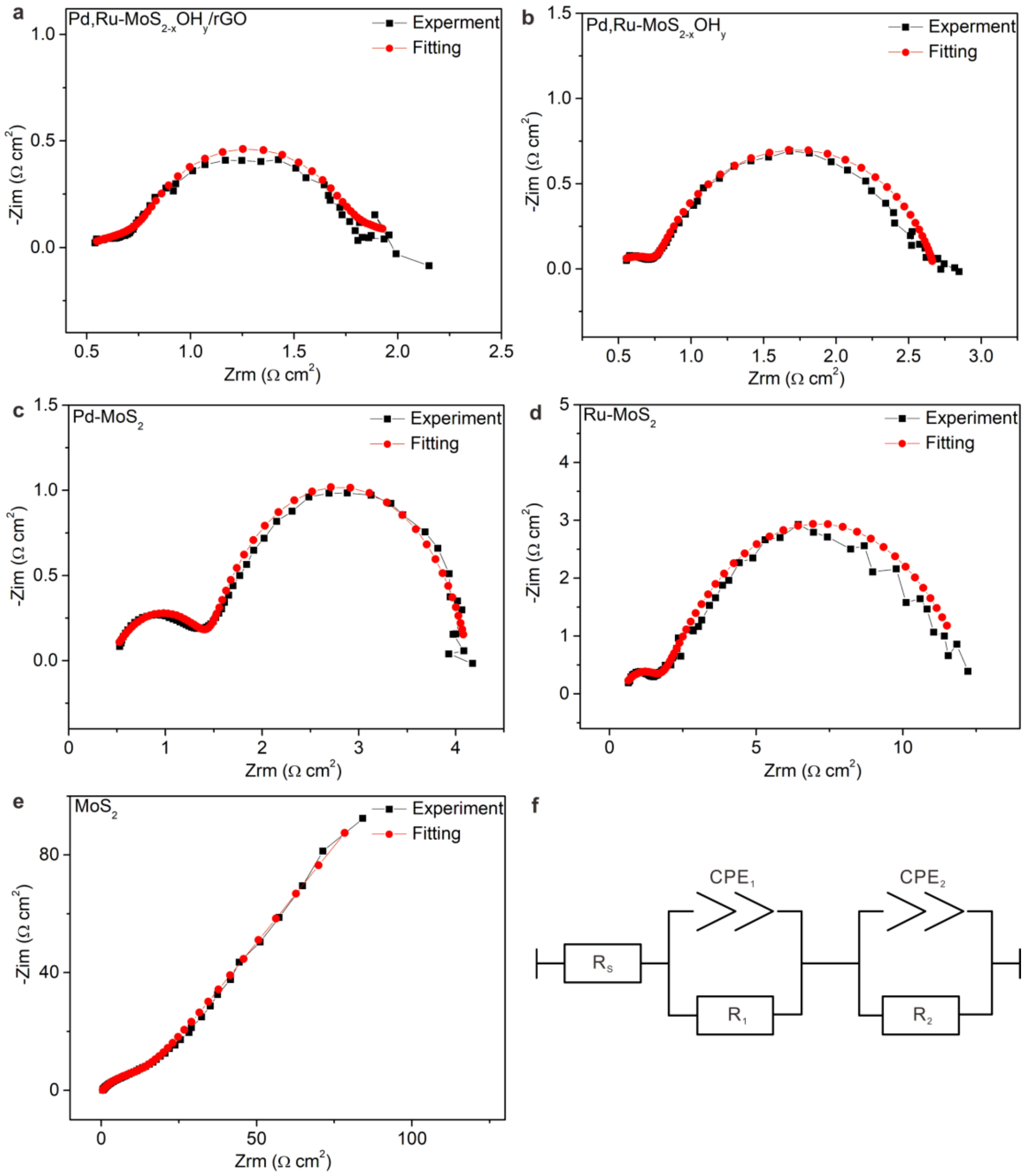

Supplementary Figure 27| EIS spectrum. a-e EIS comparison for Pd,Ru-$MoS_{2-x}OH_y$/rGO, Pd,Ru-$MoS_{2-x}OH_y$, Ru-MoS_2, Pd-MoS_2 and MoS_2 in terms of Nyquist plots. The black solid lines are experimental results, and the red dotted lines are best-fit curves. **f** Equivalent circuit models used for fitting the EIS response of HER, where Rs is the electrolyte resistance, R_1 is related to the interface resistance, R_2 denotes the charge-transfer resistance, and CPE represents the constant phase element.

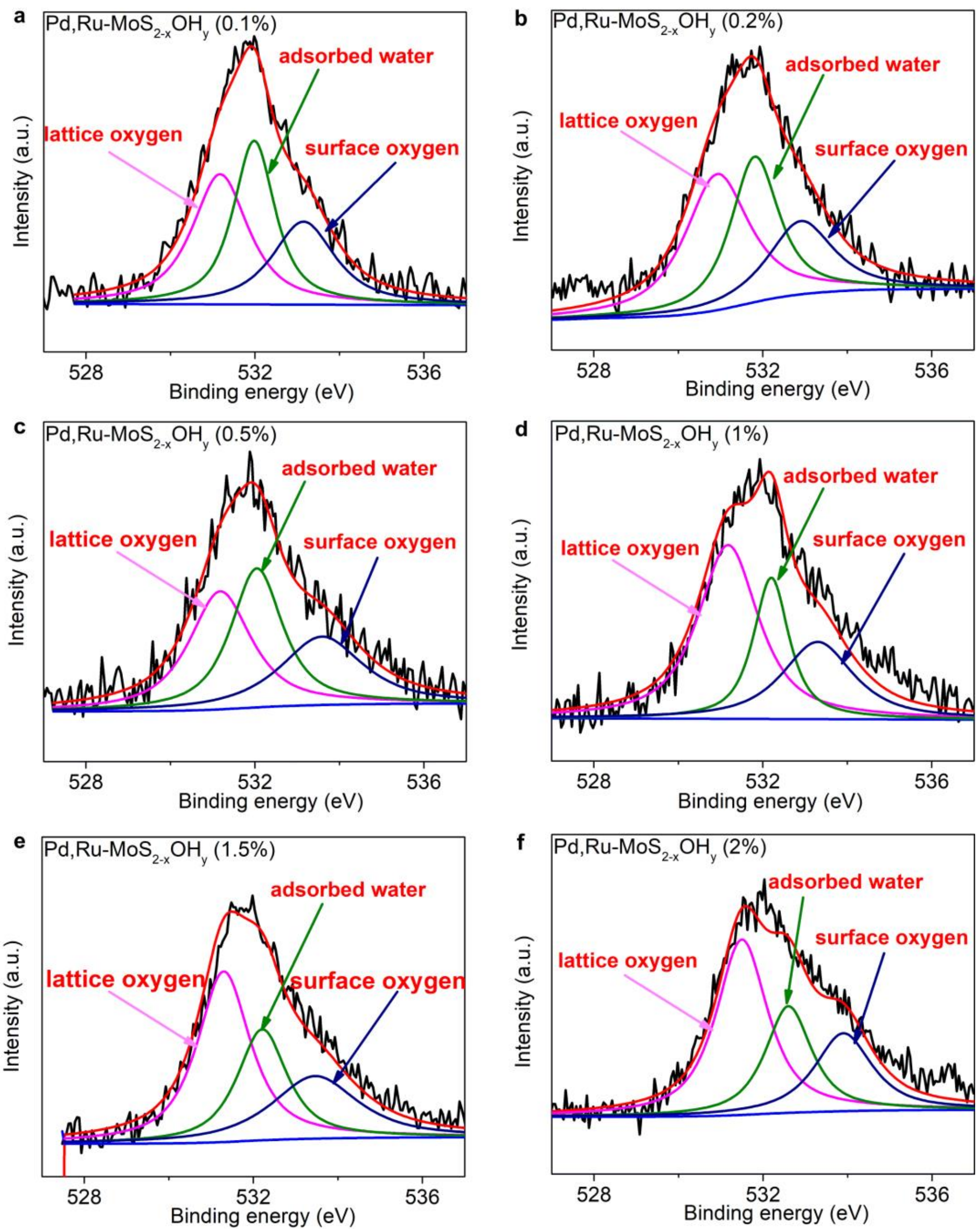

Supplementary Figure 28| XPS spectra. a High-resolution XPS results (O *1s* region) of the Pd,Ru-$MoS_{2-x}OH_y$(0.1$_{wt}$%). **b** High-resolution XPS results (O *1s* region) of the Pd,Ru-$MoS_{2-x}OH_y$(0.2$_{wt}$%). **c** High-resolution XPS results (O *1s* region) of the Pd,Ru-$MoS_{2-x}OH_y$(0.5$_{wt}$%). **d** High-resolution XPS results (O *1s* region) of the Pd,Ru-$MoS_{2-x}OH_y$(1$_{wt}$%). **e** High-resolution XPS results (O *1s* region) of the Pd,Ru-$MoS_{2-x}OH_y$(1.5$_{wt}$%). **f** High-resolution XPS results (O *1s* region) of the Pd,Ru-$MoS_{2-x}OH_y$(2$_{wt}$%).

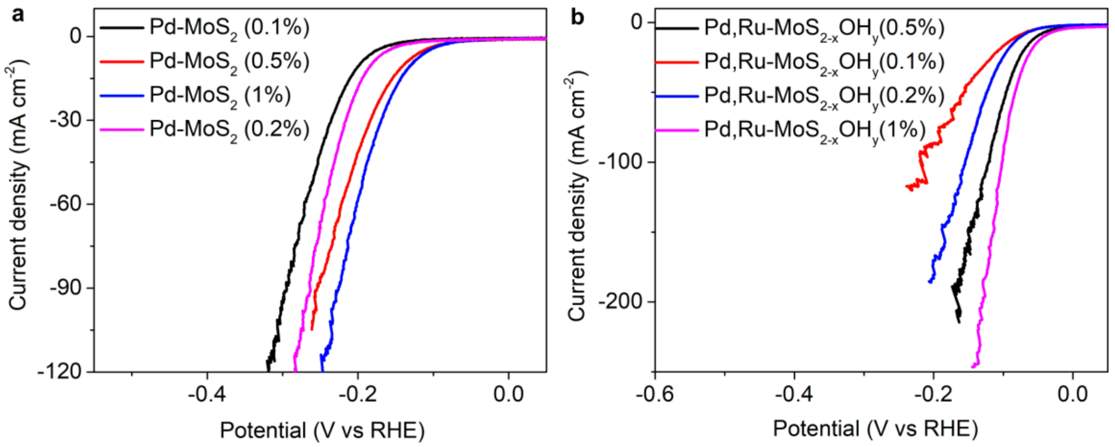

Supplementary Figure 29| HER activity in acidic media. a LSV polarization curves of Pd-MoS_2 samples with different Pd loading (0.1_{wt}%-1_{wt}%). **b** LSV polarization curves of Pd,Ru-$MoS_{2-x}OH_y$ samples with different Pd loading (0.1%-1%) and with fixed Ru loading (2.5_{wt}%).

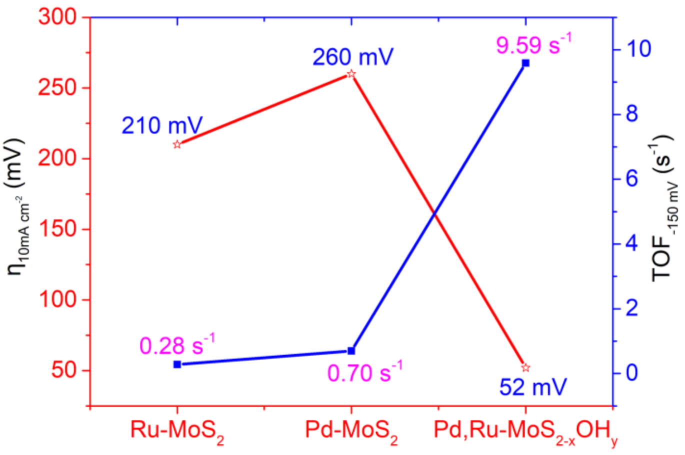

Supplementary Figure 30|TOF calculations. $TOF_{-0.15\ V}$ and $\eta_{10\ mA\ cm^{-2}}$ comparison of Ru-MoS_2, Pd-MoS_2 and Pd,Ru-$MoS_{2-x}OH_y$ samples.

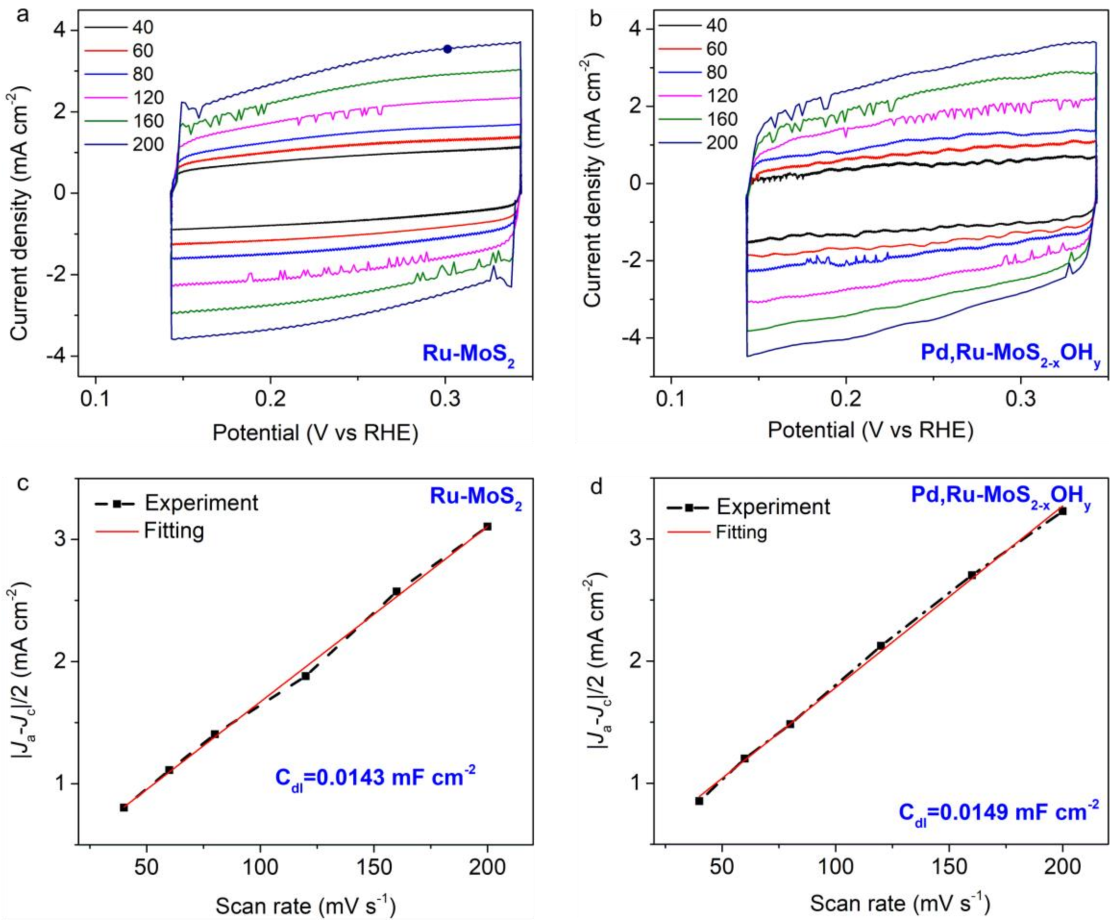

Supplementary Figure 31| double-layer measurements. a-b Cyclic voltammograms for Ru-MoS_2 and Pd,Ru-$MoS_{2-x}OH_y$ electrodes at different scan rates. **c-d** Plot showing the extraction of the double-layer capacitance (C_{dl}) for Ru-MoS_2 and Pd,Ru-$MoS_{2-x}OH_y$ electrodes.(in 1M KOH)

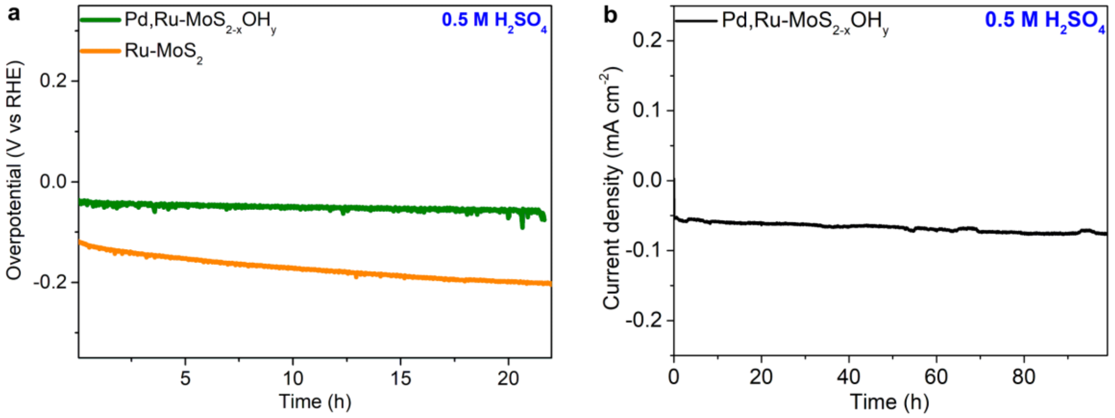

Supplementary Figure 32| HER stability in acidic media. a Chronoamperometry (CP) tests of Pd,Ru-$MoS_{2-x}OH_y$ and Ru-MoS_2 at a current density of 10 mA cm^{-2} (22 h). **b** Chronoamperometry (CP) tests of Pd,Ru-$MoS_{2-x}OH_y$ at a current density of 10 mA cm^{-2} (100 h).

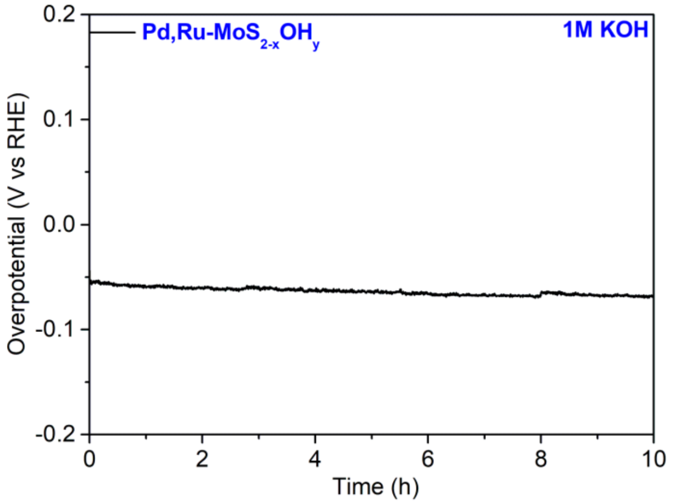

Supplementary Figure 33| HER stability in alkaline media. Long-term stability test for Pd,Ru-$MoS_{2-x}OH_y$ at a current density of 10 mA cm^{-2} in 1 M KOH.

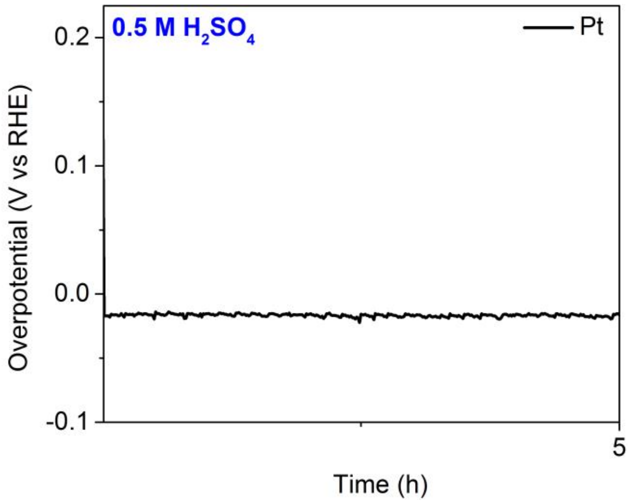

Supplementary Figure 34| HER Stability. Galvanostatic experiments of Pt at a current density of 10 mA cm^{-2}**.**

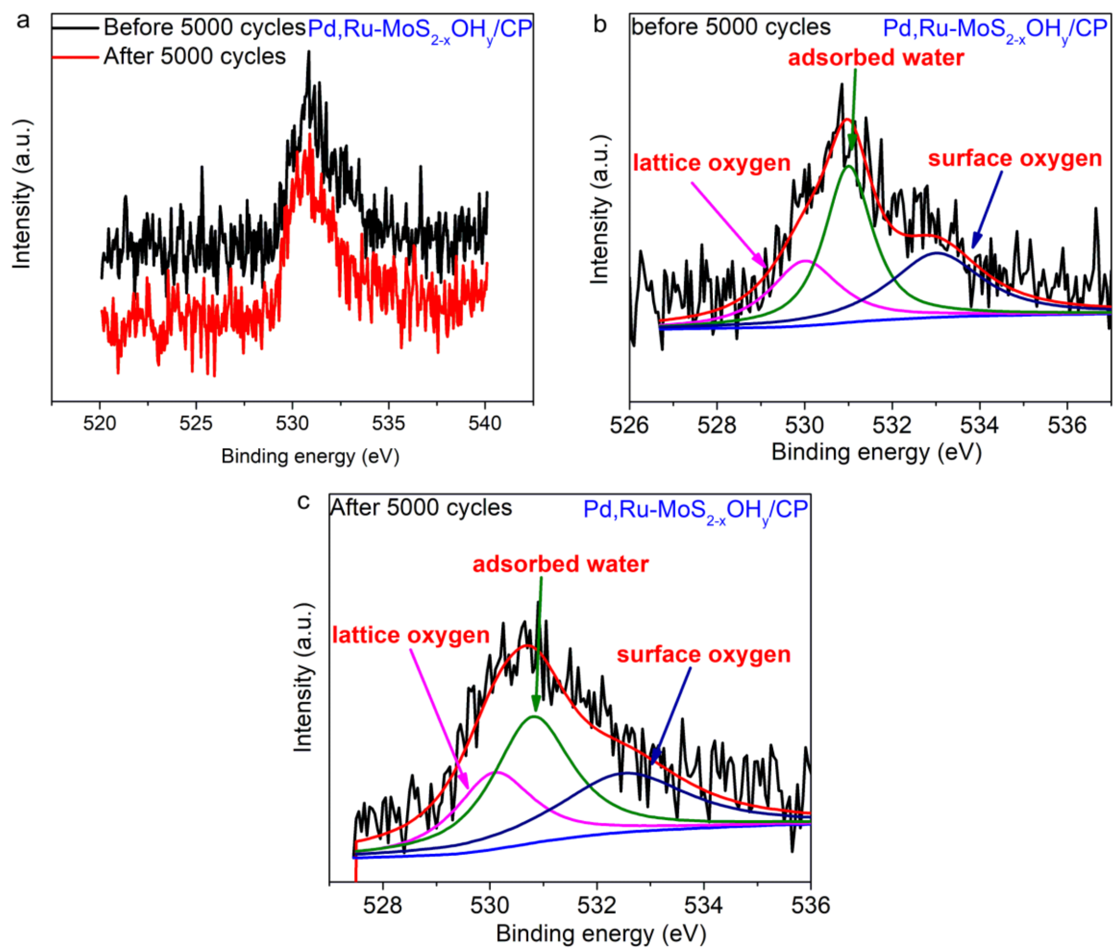

Supplementary Figure 35| XPS spectra. a High-resolution XPS results (O *1s* region) of the Pd,Ru-$MoS_{2-x}OH_y$/CP samples before and after 5000 cycles. **b** High-resolution XPS results (O *1s* region) of the Pd,Ru-$MoS_{2-x}OH_y$/CP sample before 5000 cycles. **c** High-resolution XPS results (O 1s region) of the Pd,Ru-$MoS_{2-x}OH_y$/CP sample after 5000 cycles.

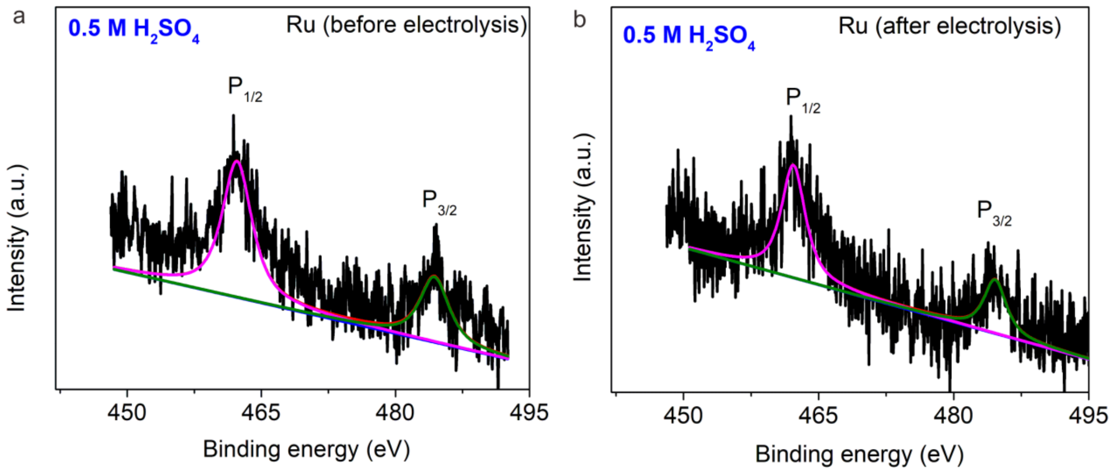

Supplementary Figure 36| XPS spectra. a High-resolution XPS results (Ru 2p region) of the Pd,Ru-$MoS_{2-x}OH_y$ before electrolysis at 10 mA cm^{-2} for 10 h. **b** High-resolution XPS results (Ru 2p region) of the Pd,Ru-$MoS_{2-x}OH_y$ after electrolysis at 10 mA cm^{-2} for 10 h.

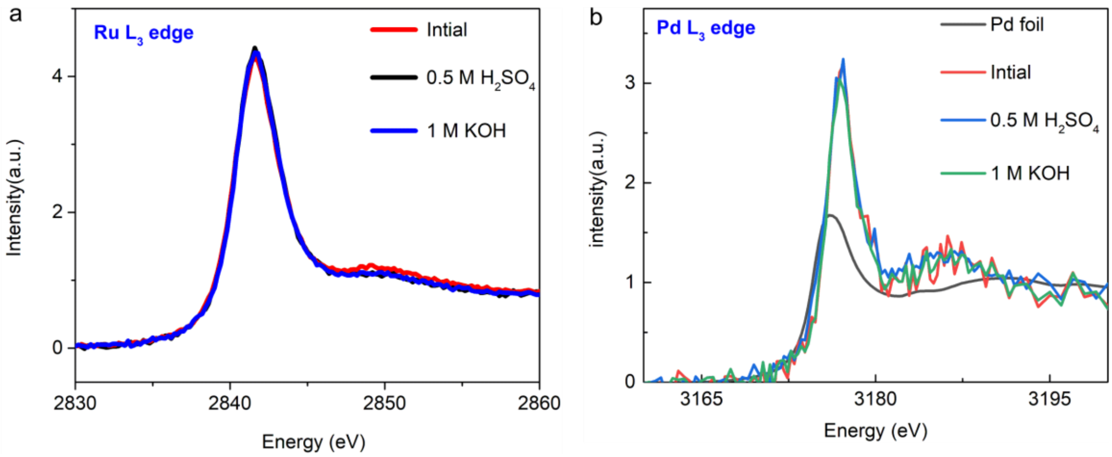

Supplementary Figure 37| EXAFS spectra. a Ru L_3-edge XANES spectra of the Pd,Ru-$MoS_{2-x}OH_y$. **b** Pd L_3-edge XANES spectra of the Pd,Ru-$MoS_{2-x}OH_y$, and the XANES data of the reference standards of Pd foil .

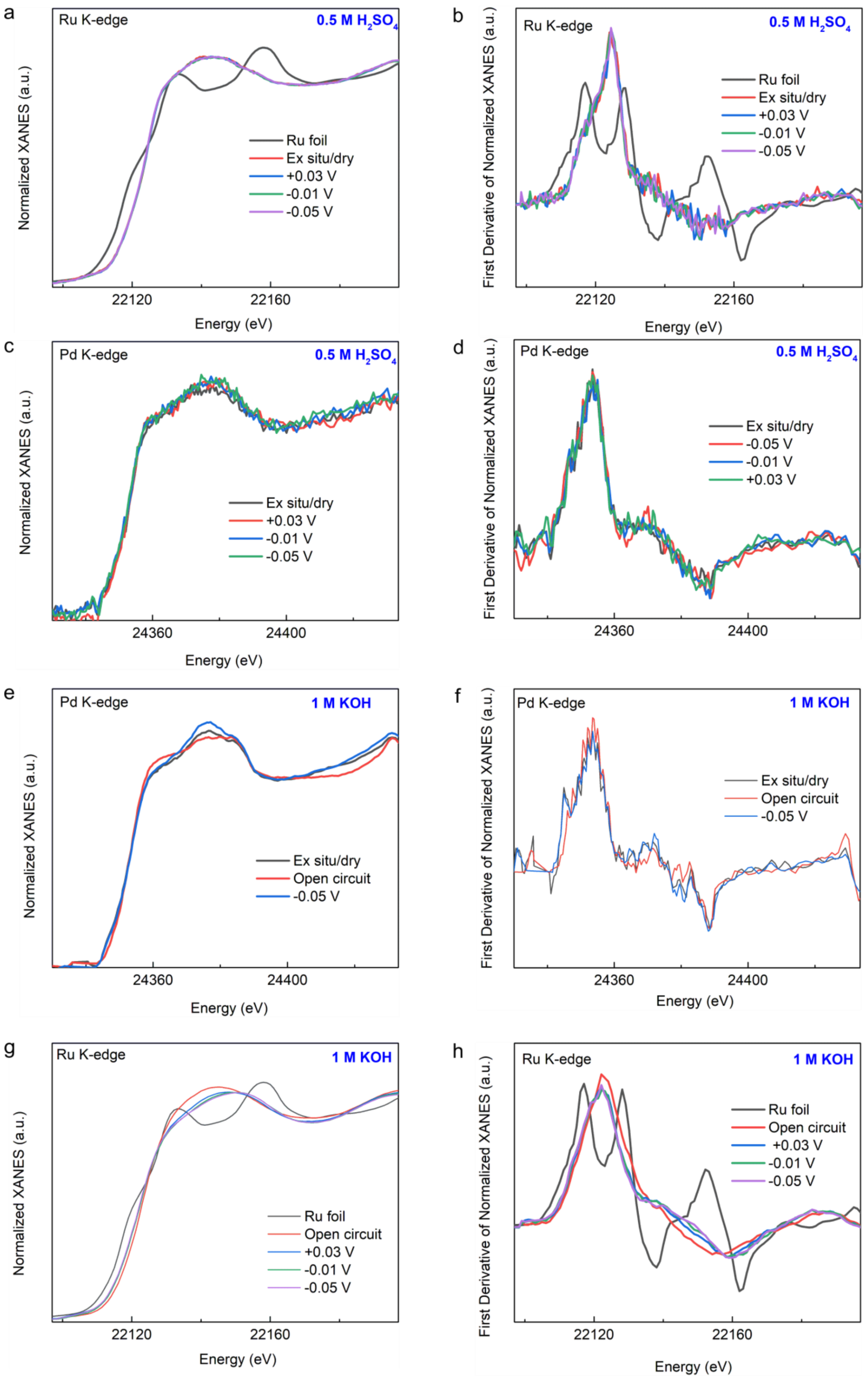
a
Ru K-edge
0.5 M H_2SO_4
Ru foil
Ex situ/dry
+0.03 V
-0.01 V
-0.05 V
Normalized XANES (a.u.)
22120
22160
Energy (eV)
b
Ru K-edge
0.5 M H_2SO_4
Ru foil
Ex situ/dry
+0.03 V
-0.01 V
-0.05 V
First Derivative of Normalized XANES (a.u.)
22120
22160
Energy (eV)
c
Pd K-edge
0.5 M H_2SO_4
Ex situ/dry
+0.03 V
-0.01 V
-0.05 V
Normalized XANES (a.u.)
24360
24400
Energy (eV)
d
Pd K-edge
0.5 M H_2SO_4
Ex situ/dry
-0.05 V
-0.01 V
+0.03 V
First Derivative of Normalized XANES (a.u.)
24360
24400
e
Pd K-edge
1 M KOH
Ex situ/dry
Open circuit
-0.05 V
Normalized XANES (a.u.)
24360
24400
Energy (eV)
f
Pd K-edge
1 M KOH
Ex situ/dry
Open circuit
-0.05 V
First Derivative of Normalized XANES (a.u.)
24360
24400
Energy (eV)
g
Ru K-edge
1 M KOH
Ru foil
Open circuit
+0.03 V
-0.01 V
-0.05 V
Normalized XANES (a.u.)
22120
22160
Energy (eV)
h
Ru K-edge
1 M KOH
Ru foil
Open circuit
+0.03 V
-0.01 V
-0.05 V
First Derivative of Normalized XANES (a.u.)
22120
22160
Energy (eV)

Supplementary Figure 38| Operando XANES spectra. a Operando XANES spectra recorded at the Ru K-edge of Pd,Ru-$MoS_{2-x}OH_y$ (in 0.5 M H_2SO_4) . **b** The first-derivative of XANES spectra in the left figure (**a**). **c** Operando XANES spectra recorded at the Ru K-edge of Pd,Ru-$MoS_{2-x}OH_y$ (1 M KOH). **d** The first-derivative of XANES spectra in the left figure (**c**). **e** Operando XANES spectra recorded at the Pd K-edge of Pd,Ru-$MoS_{2-x}OH_y$ (in 0.5 M H_2SO_4). **f** The first-derivative of XANES spectra in the left figure (**e**). **g** Operando XANES spectra recorded at the Pd K-edge of Pd,Ru-$MoS_{2-x}OH_y$(in 1 M KOH). **h** The first-derivative of XANES spectra in the left figure (**g**).

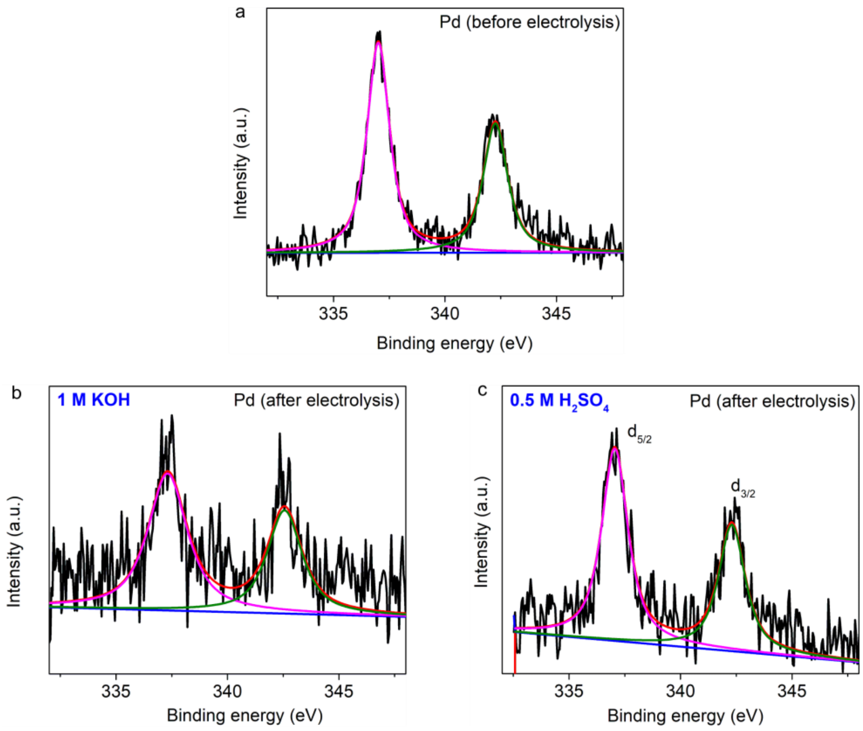

Supplementary Figure 39| XPS spectra. a High-resolution XPS results (Pd 3d region) of the Pd,Ru-$MoS_{2-x}OH_y$ before electrolysis at 10 mA cm^{-2} for 10 h. **b** High-resolution XPS results (Pd 3d region) of the Pd,Ru-$MoS_{2-x}OH_y$ after electrolysis at 10 mA cm^{-2} for 10 h (1 M KOH). **c** High-resolution XPS results (Pd 3d region) of the Pd,Ru-$MoS_{2-x}OH_y$ after electrolysis at 10 mA cm^{-2} for 10 h (0.5 M H_2SO_4).

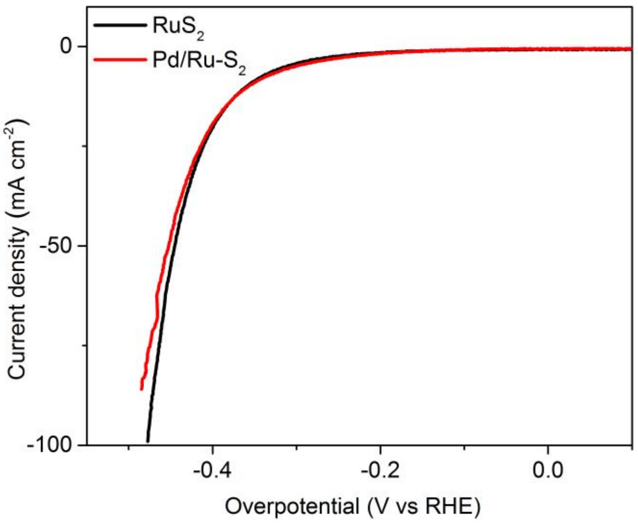

Supplementary Figure 40| HER activity. The HER activity of RuS_2 and Pd/Ru-S_2.

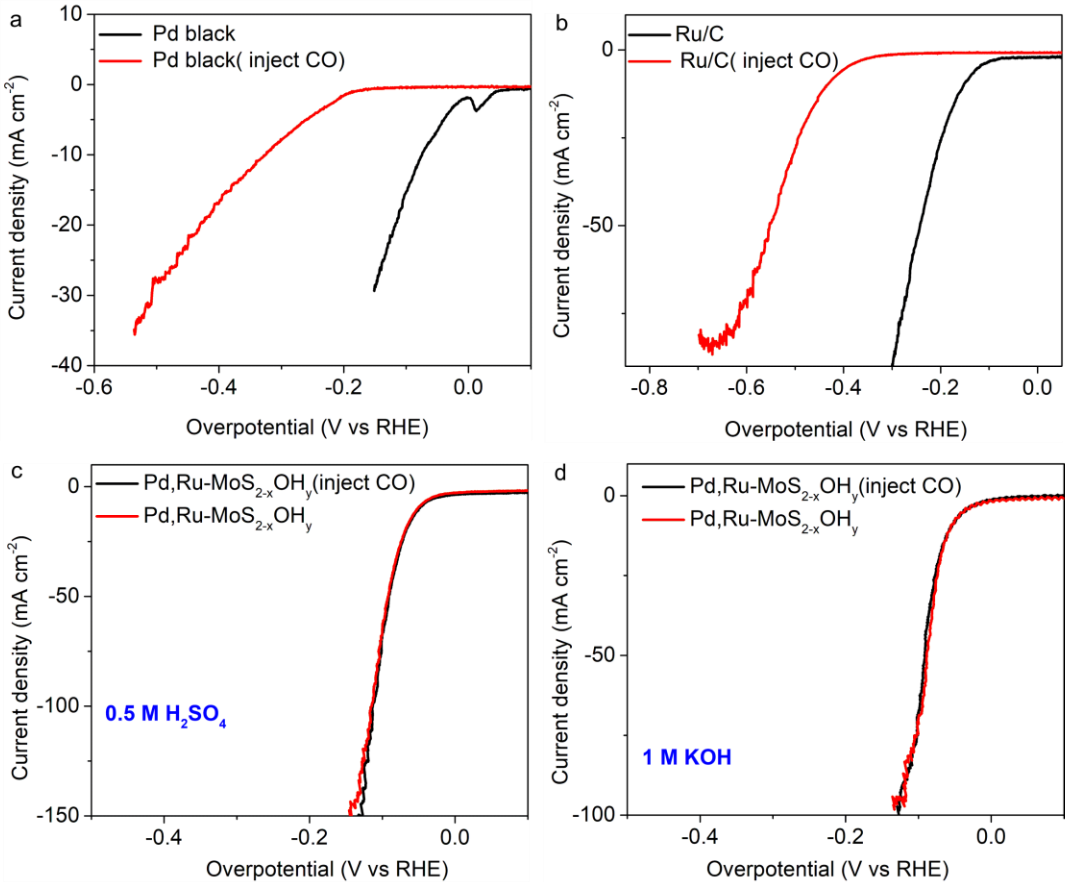

Supplementary Figure 41| tolerance experiments. a CO tolerance experiment of the commercial Pd black. **b** CO tolerance experiment of the commercial Ru/C. **c** CO tolerance experiment of the Pd,Ru-$MoS_{2-x}OH_y$ (0.5 M H_2SO_4). **d** CO tolerance experiment of the Pd,Ru-$MoS_{2-x}OH_y$ (1 M KOH).

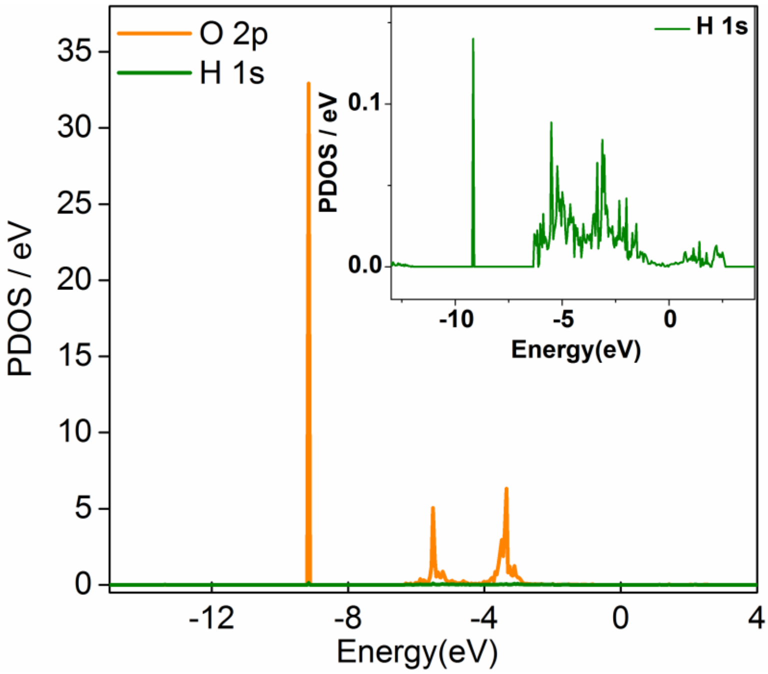

Supplementary Figure 42| DFT calculations. pDOS curves for H_2O adsorption on OH sites.

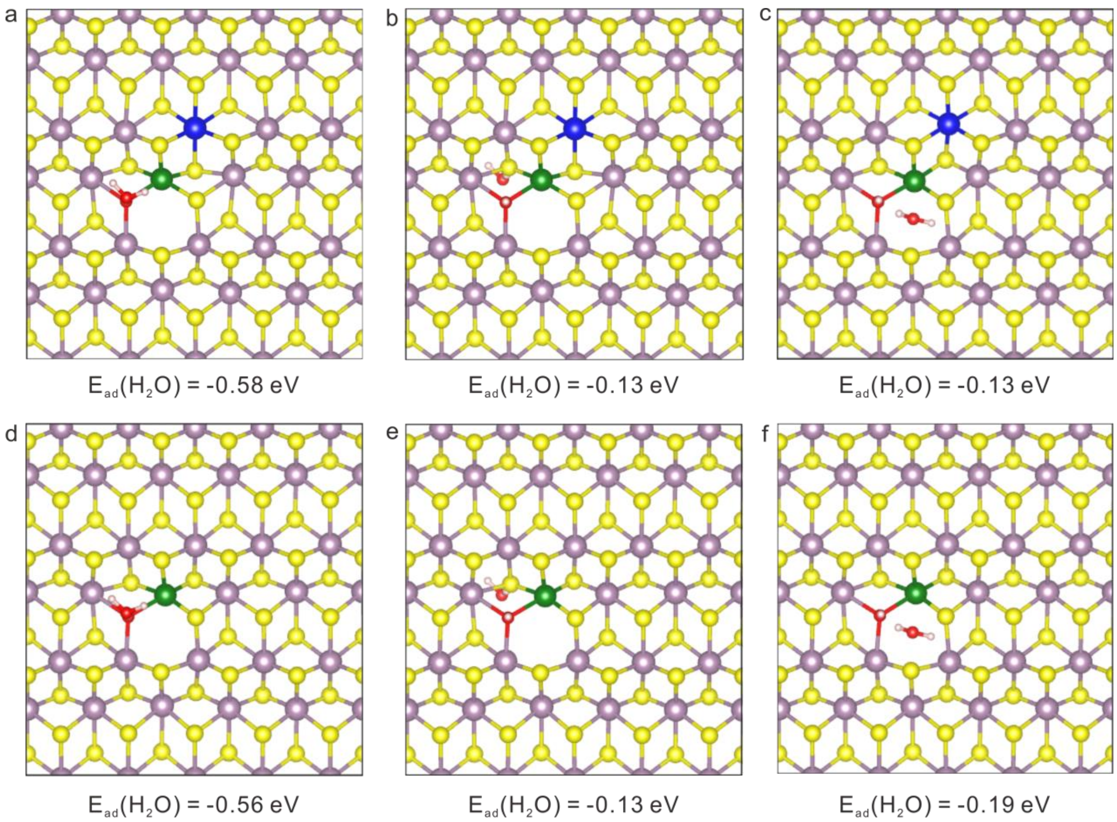

Supplementary Figure 43|Water adsorption energies on different sites. a The water adsorption energies on the OH sites of Pd,Ru-$MoS_{2-x}OH_y$. **b** The water adsorption energies on the S sites of Pd,Ru-$MoS_{2-x}OH_y$. **c** The water adsorption energies on the SVs sites of Pd,Ru-MoS_2. **d** The water adsorption energies on the OH sites of Ru-$MoS_{2-x}OH_y$. **e** The water adsorption energies on the S sites of Ru-$MoS_{2-x}OH_y$. **f** The water adsorption energies on the SVs sites of Ru-$MoS_{2-x}OH_y$. (When Pd and Ru atoms are not coordinated with same S atoms, the effect of Ru and Pd co-doping is similar to Ru-doping, here, we constructed the doping model by Ru to perform the correlative DFT calculations)

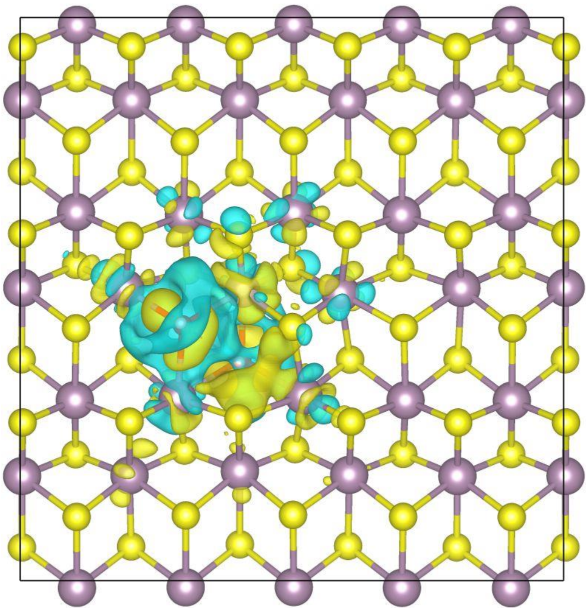

Supplementary Figure 44| DFT calculations. Electron density difference plot of the OH adsorption structure. Yellow contours indicate electron accumulation and light green contours denote electron deletion.

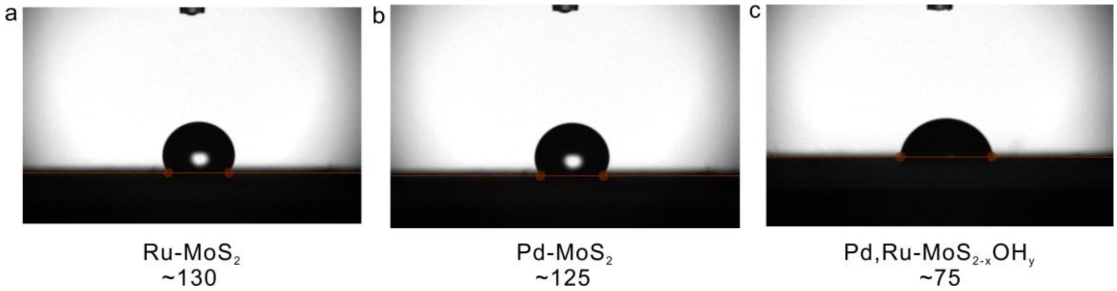

Supplementary Figure 45| Contact angels analysis. a contact angel of Ru-MoS_2. **b** contact angel of Pd-MoS_2. **c** contact angel of Pd,Ru-$MoS_{2-x}OH_y$.

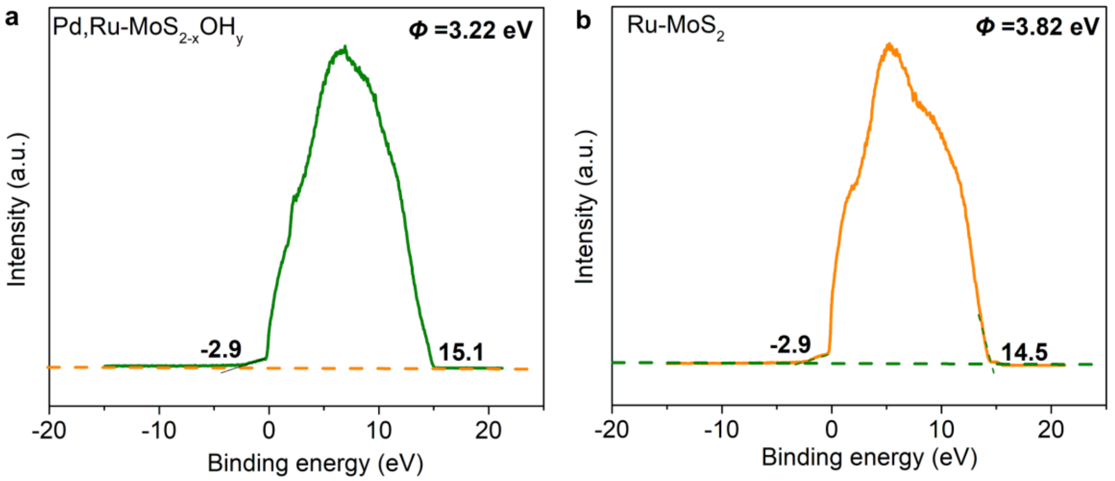

Supplementary Figure 46| UPS spectra.UPS spectrum of Pd,Ru-$MoS_{2-x}OH_y$ and Ru-MoS_2.

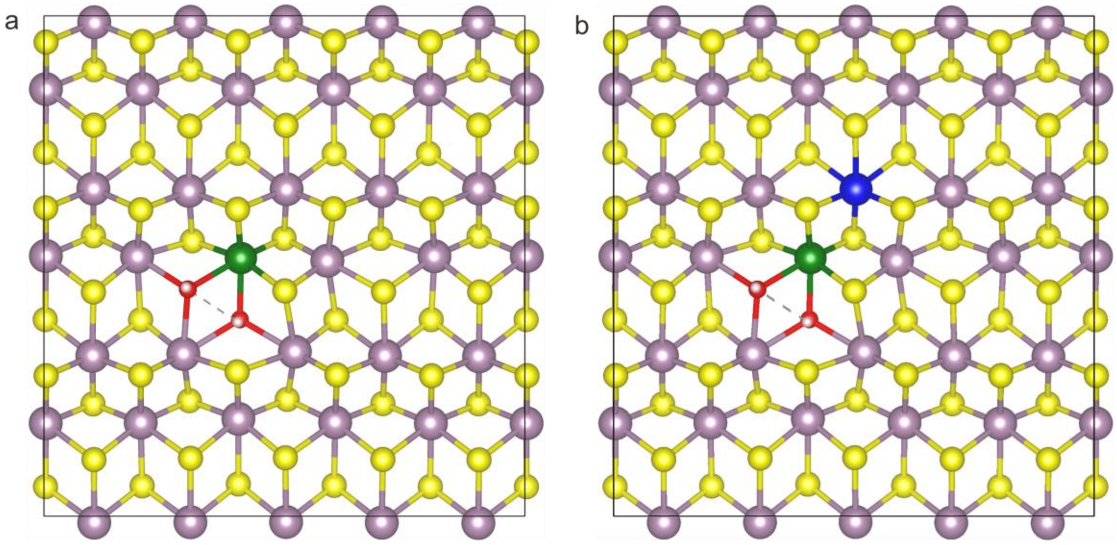

Supplementary Figure 47| DFT calculations. Calculated adsorption energies of OH on the sites of Pd,Ru-$MoS_{2-x}OH_y$ (**a**: − 2.8 eV; **b**: −2.54 eV). (When Pd and Ru atoms are not coordinated with same S atoms, the effect of Ru and Pd co-doping is similar to Ru-doping, here, we constructed the doping model by Ru to perform the correlative DFT calculations)

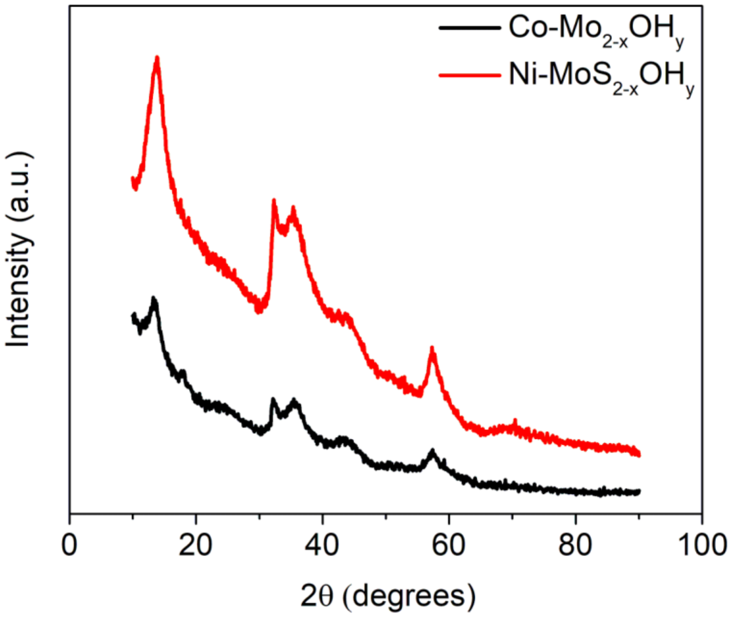

Supplementary Figure 48| XRD patterns. XRD patterns of Co-$MoS_{2-x}OH_y$ and Ni-$MoS_{2-x}OH_y$ samples.

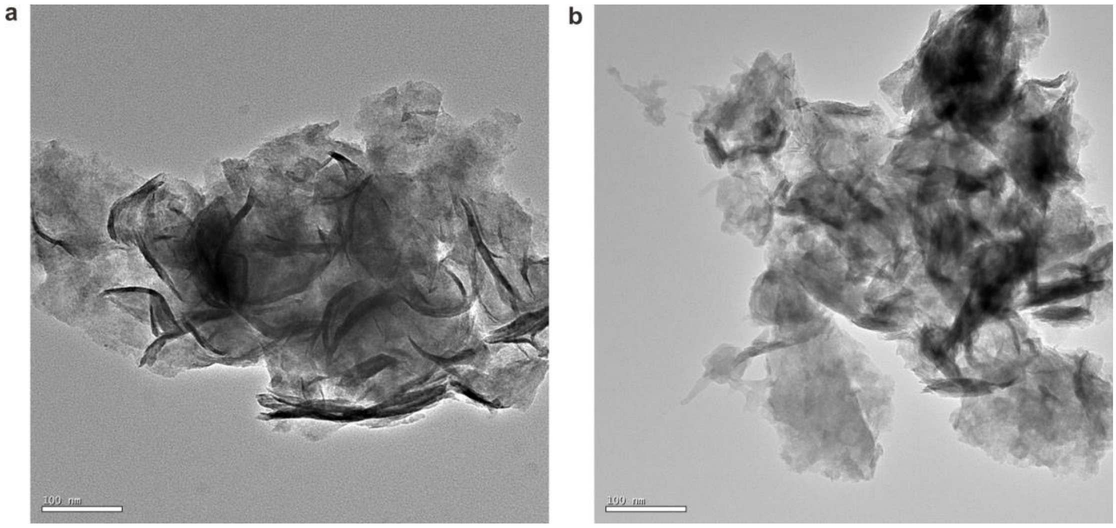

Supplementary Figure 49| TEM patterns. a TEM of Ni-$MoS_{2-x}OH_y$. **b** TEM of Co-$MoS_{2-x}OH_y$.

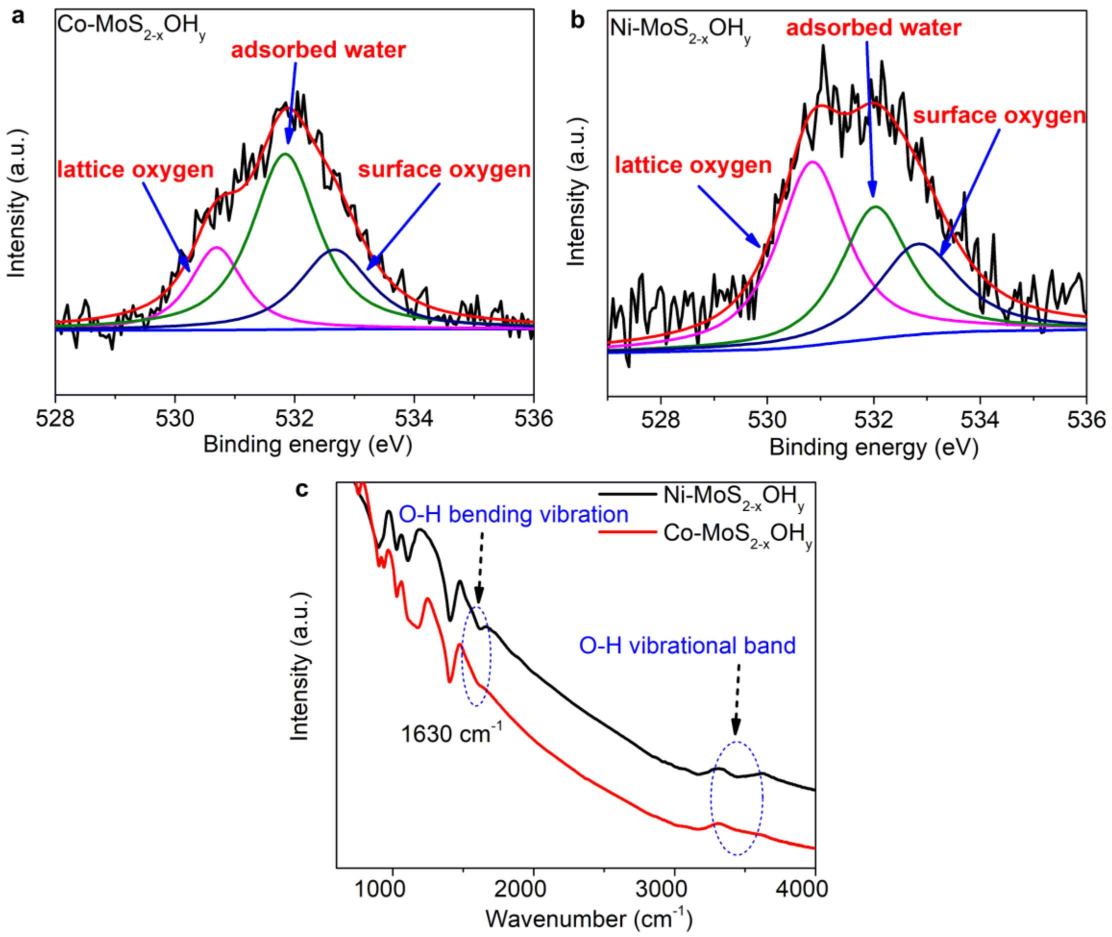

Supplementary Figure 50|XPS patterns and FITR pattern. a High-resolution XPS results (O *1s* region) of the Co-$MoS_{2-x}OH_y$. **b** High-resolution XPS results (O *1s* region) of the Ni-$MoS_{2-x}OH_y$. **c** FTIR spectra of Ni-$MoS_{2-x}OH_y$ and Co-$MoS_{2-x}OH_y$.

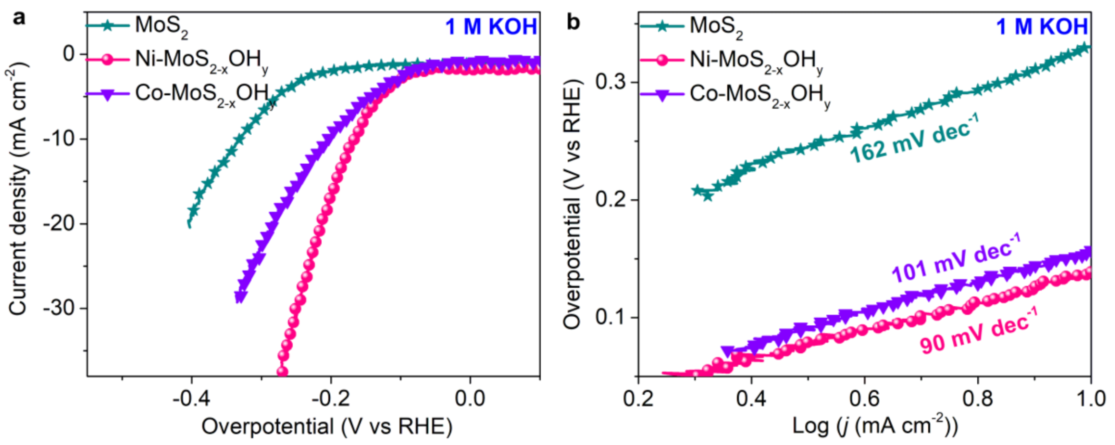

Supplementary Figure 51| HER activity. a LSV polarization curves of MoS_2, Co-$MoS_{2-x}OH_y$ and Ni-$MoS_{2-x}OH_y$. (with iR correction) in 1M KOH. **b** Tafel plots derived from the results given in Fig. 33 **a.**

Supplementary Table 1. Ru K-edge EXAFS curves fitting parameters.

Sample	path	*N*	*R*(Å)	σ^2(10^{-3} Å^2)	ΔE_0(eV)	*R*-factor
Ru-MoS_2	Ru-S	6.2	2.33	5	-1.7	0.010
	Ru-Mo	0.9	2.78	5.0	1.5	
Pd,Ru-$MoS_{2-x}OH_y$	Ru-S	4.5	2.31	6.6	1.5	0.013
	Ru-O	1.0	2.07	3.0	-1.2	
	Ru-Mo	0.4	2.78	3.0	1.5	

N, coordination number; *R*, distance between absorber and backscatter atoms; σ^2, Debye-Waller factor; ΔE_0, inner potential correction accounting for the difference in the inner potential between the sample and the reference compound.

Supplementary Table 2. Pd K-edge EXAFS curves fitting parameters.

Sample	Path	*N*	*R*(Å)	σ^2(10^{-3} Å^2)	ΔE_0 eV)	*R*-facto
Pd,Ru-$MoS_{2-x}OH_y$	Pd-S	3.9	2.31	2.7	-4.4	0.006
	Pd-Mo1	0	2.77	3.0	10.0	
	Pd-Mo2	2.2	3.04	3.0	17.9	

N, coordination number; *R*, distance between absorber and backscatter atoms; σ^2, Debye-Waller factor; $\Delta E_{0,}$ inner potential correction accounting for the difference in the inner potential between the sample and the reference compound.

Supplementary Table 3. Mo K-edge EXAFS curves fitting parameters.

Sample	Path	N	R(Å)	σ^2(10^{-3} Å^2)	ΔE_0(eV)	R-factor
MoS_2	Mo-S	4.5	2.3	7.6	0.4	0.014
	Mo-Mo	1.5	2.79	8.8	7.1	

N, coordination number; R, distance between absorber and backscatter atoms; σ^2, Debye-Waller factor; ΔE_0, inner potential correction accounting for the difference in the inner potential between the sample and the reference compound.

Supplementary Table 4. ICP-OES results of dissolved S, Mo, Ru and Pd ions in solution after this redox process.

Dissolved ions into the solution (μg mL^{-1})	Mo	S	Pd	Ru
Ru-MoS_2	0	0	-	0
Pd,Ru-$MoS_{2-x}OH_y$	271.0	229.6	0	0

We mixed the Ru-MoS_2 sample with Pd (II) solutions for reaction. The leaching of Mo ions and S ions into the solution was also detected by ICP-OES, further verify our reported spontaneous interfacial redox doping mechanism. Moreover, no Ru (under the detection limit) was obtained in the solution. These results exclude the possibility of Pd displace Ru sites.

Supplementary Table 5. Mo K-edge EXAFS curves fitting parameters.

Sample	path	N	R(Å)	σ^2(10^{-3} Å^2)	ΔE_0(eV)	R-factor
Ru-MoS_2	Mo-S	4.3	2.40	7.3	-1.3	0.020
	Mo-Mo1	0.7	2.80	11.4	8.7	
	Mo-Mo2	0.6	3.14	6.4	13.1	
Pd,Ru-$MoS_{2-x}OH_y$	Mo-S	3.8	2.40	4.7	0.9	0.016
	Mo-O	0.3	2.13	5.0	0.9	
	Mo-Mo1	0.8	2.82	8.8	10.0	
	Mo-Mo2	0.9	3.18	2.4	10.0	

N, coordination number; R, distance between absorber and backscatter atoms; σ^2, Debye-Waller factor; $\Delta E_{0,}$ inner potential correction accounting for the difference in the inner potential between the sample and the reference compound.

Supplementary Table 6| Comparison of HER activity for Pd,Ru-$MoS_{2-x}OH_y$ with other MoS_2-based electrocatalysts in acidic media.

Catalyst	Electrolytes	η (mV vs RHE) for j = -10 mA cm^{-2}
$CoMoS_3$-PHSMS[1]	0.5 M H_2SO_4	171
MoS_2 flakes<br>MoS_2 films[2]	0.5 M H_2SO_4	~250<br>~175
P-1T-MoS_2<br>P-2H-MoS_2	0.5 M H_2SO_4	153<br>218
P-2H-MoS_2+S<br>1T-MoS_2<br>2H-MoS_2[3]		257<br>203<br>343
V-MoS_2(EC)[4]	0.5 M H_2SO_4	320
MoS_2-BP[5]	0.5 M H_2SO_4	85
MoSSe[6]	0.5 M H_2SO_4	300
Pt-MoS_2[7]	0.1 M H_2SO_4	~140
SV-MoS_2<br>V-MoS_2[8]	0.5 M H_2SO_4	170<br>250
Edge ox 1T MoS_2[9]	0.5 M H_2SO_4	~200
SL-MoS_2-CNTS[10]	0.5 M H_2SO_4	~220
M-MoS_2[11]	0.5 M H_2SO_4	175

$MoS_2/CoSe_2$[12]	0.5 M H_2SO_4	68
2H MoS_2 flakes[13]	0.5 M H_2SO_4	286
Zn-MoS_2[14]	0.5 M H_2SO_4	150

Supplementary Table 7. Comparison of the exchange current density(j_0) and turnover frequency (TOF) for Pd,Ru-$MoS_{2-x}OH_y$ with Pd-MoS_2 and Ru-MoS_2 in 0.5 M H_2SO_4.

	Pd,Ru-$MoS_{2-x}OH_y$	Pd-MoS_2	Ru-MoS_2	MoS_2
J_0 (mA cm^{-2})	2.884	0.302	0.813	0.025
$TOF_{0\ V}$ (s^{-1})	0.892	0.093	0.048	-
$TOF_{-0.1\ V}$ (s^{-1})	17.739	0.877	0.369	-
$TOF_{-0.13\ V}$ (s^{-1})	34.582	1.555	0.624	-

Supplementary Table 8. The EIS fitting results of Pd,Ru-$MoS_{2-x}OH_y$/rGO, Pd,Ru-$MoS_{2-x}OH_y$, Ru-MoS_2, Pd-MoS_2 and MoS_2.

Sample	R_{ct} (Ω cm^2)
MoS_2	386.54
Ru-MoS_2	10.64
Pd-MoS_2	2.64
Pd,Ru-$MoS_{2-x}OH_y$	1.89
Pd,Ru-$MoS_{2-x}OH_y$/rGO	0.91

Supplementary Table 9. Summary of element ratio Pd,Ru-$MoS_{2-x}OH_y$.

Pd,Ru-$MoS_{2-x}OH_y$ samples	Pd (wt%)	Ru (at%)	Mo (at%)	S (at%)	OH (at%)
1	0.1	1.1	21.1	35.49	1.20
2	0.2	1.25	20.1	34.3	1.69
3	0.5	1.14	20.86	34.96	2.00
4	1.0	1.18	19.32	34.02	3.68
5	1.5	1.21	19.27	33.99	5.21
6	2.0	1.20	18.13	33	5.90

Supplementary Table 10. Summary of current density achieved at η=140 mV as a function of Pd content in Pd, Ru-$MoS_{2-x}OH_y$ and Pd-MoS_2.

wt(%) / *J* (mA cm^{-2})	0.1%	0.2%	0.5%	1%
Pd,Ru-$MoS_{2-x}OH_y$	39.07	75.42	136.63	245.66
Pd-MoS_2	1.55	2.98	9.68	14.74

Supplementary Note 1: TOF calculation

The TOF was calcualted according the formula:

$$TOF = \frac{Total\ \ number\ H_2\ atoms\ per\ second}{Total\ number\ of\ active\ sites\ per\ unit\ are} = \frac{j/(2\times q)}{N} \qquad \textbf{Supplementary Equation 1}$$

Where q = 1.6×10^{-19} C is the elementary charge, and 2 accounts for 2 H atoms per H_2 molecule.

The DFT calculations suggest that the S atoms near the Pd dopants in the MoS_2 are the prior adsorption sites. In the following we use the number of the sulfur vacancies and the S atoms near the Pd dopants in the MoS_2 as the number of active sites, which can be estimated from the number of Pd.

Supplementary Note 2: the effect of doped Pd(or Ru)

We have actually used a combination of DFT calculation and experimental techniques to confirm the real active sites and validate that MoS_2, instead of Pd or Ru, is the true catalyst. In addition, control experiments were carried out using CO poisoning technique to exclude the possibility of Pd/Ru being the active sites. We will show the evidences in sequences:

- Firstly, the Pd-S and Ru-S coordination numbers are 4.5 and 5.5 in the final sample, suggesting a similar coordination environment to Pd/Ru-S_2 and RuS_2 samples. We therefore tested the HER performances of these samples, and the results are shown in Supplementary Fig. 40. Notably, neither of the samples show appreciable HER activity, demonstrating that the Pd and Ru atoms, in their oxidation status, are lacking HER catalytic property in contrast to their metallic states.
- Secondly, the CO poisoning experiments were carried out according to the reviewer's suggestion. Supplementary Fig. 41 shows the CO poisoning experiment results via bubbling CO into the electrolyte during recording LSVs. Specifically, Pd,Ru-$MoS_{2-x}OH_y$ shows no observable performance decay after CO injection. In contrast, the injection of CO caused huge performance

decay of the commercial Pd/C and Ru/C. Thus, it is confirmed that the active sites in Pd,Ru-$MoS_{2-x}OH_y$ are distinct from that of metallic Pd and Ru. Combining the fact that Pd/Ru-S_2 and RuS_2 samples are not active towards HER, we thus claim that the Pd and Ru sites are not the active sites in the Pd,Ru-$MoS_{2-x}OH_y$ material.

- Thirdly, if Pd in the Pd,Ru-$MoS_{2-x}OH_y$ material is the active site, the HER catalytic activity of the material will increase with the increase of Pd content in the sample. However, as shown in our previously reported work[1], an optimum value of Pd doping is observed in the Pd-MoS_2, where Pd doping content beyond 10% results in performance decay of the catalysts. This phenomenon can only be interpreted if Pd atoms are not the active sites, who instead function by activating the MoS_2 basal plane through the introduction of sulfur vacancies (SVs) and by activate the adjacent S atoms (Pd-S*-Mo). This is explained in the following through DFT calculations.
- Fourthly, we carried out DFT calculations to probe into the adsorption properties of the materials through calculating the free energy for atomic hydrogen adsorption(ΔG_{H*}) of different sites. The Pd sites themselves were calculated to be inert as H does not form a very stable adsorption structure on Pd atop site[1]. However, the ΔG_{H*} of the S atop site adjacent to Pd (Pd–S*–Mo) in the Pd–MoS_2 exhibits an almost thermoneutral value of −0.02 eV[1], reaching a ΔG_{H*} comparable to that of platinum. Therefore, Pd atoms are not the active sites, and instead, they function by activating the Pd bonded sulfur atoms to exhibit optimal ΔG_{H*} atoms (Pd–S*–Mo).
- Finally, we conducted further XPS and (in situ) XANES investigations to exclude the possibility of generating Pd/Ru metallic particles during the operation, which may serve as true active sites. Supplementary Fig. 36-38 show the post XPS and XANES results of the Pd,Ru-$MoS_{2-x}OH_y$ sample after electrolysis. Notably, neither the content nor the valance state of Pd was altered for

the post test sample according to the XPS, suggesting that Pd is firmly integrated into the MoS_2 backbone and highly stable under electrolytic conditions (Supplementary Fig. 39b-c). Meanwhile, The Pd L_3-edge XANES results (Supplementary Fig. 37) demonstrate no change in the white line resonance strength in comparison to the Pd,Ru-$MoS_{2-x}OH_y$ sample before the electrolysis test, thereby suggesting that the average valence of Pd sites is not changed. Furthermore, in-situ XANES spectra were adopted in the revised manuscript to supplement our claim, as is shown in Supplementary Fig. 38. Using operando XANES spectra at the Pd K-edge, no shift of the absorption edge is observed with cathode potential varying between 0 and −0.1 V (vs. RHE), implying no change in the Pd average valence state in Pd,Ru-$MoS_{2-x}OH_y$ sample during the HER process. Therefore, in situ XAS provides most direct evidence to support our claim that Pd will not be reduced under HER conditions.

To here, it is clear that real catalyst is MoS_2, not Pd sites.

Supplementary Note 3:

In light of these findings, we further incorporated other metal oxide sites (M-OH, M= Ni, Co) into the basal plane of MoS_2 to identify the effect of incorporating M-OH sites on the HER activity of MoS_2 catalysts. The X-ray diffraction (XRD) pattern (Supplementary Fig. 48) shows that as-synthesized samples can be indexed as MoS_2 and no other crystal phases appearing. This indicates that the homogenous incorporation of metal oxide ions into the lattice of MoS_2. Moreover, the structure and morphology of MoS_2 modified by M-OH sites (M=Co, Ni) will not be changed compared with the pure MoS_2. (Supplementary Fig. 49) It should be noted that the O 1s spectrum of the Ni-$MoS_{2-x}OH_y$ and Co-$MoS_{2-x}OH_y$ can also be deconvoluted into three peaks at approximately 529.6, 534.1 and 531.0 eV,

corresponding to adsorbed H_2O, surface oxygen and M-OH (M=Ni, Co) species, respectively. (Supplementary Fig. 50a-b) FTIR results (Supplementary Fig. 50c) of Ni-$MoS_{2-x}OH_y$ and Co-$MoS_{2-x}OH_y$ samples observed the signal of O-H vibrational bands peak at 3,000–3,500 cm^{-1} and O-H bending vibration peak at 1630 cm^{-1}, which further the presence of Ni (Co)-OH. We estimated the HER catalytic activity of Ni–$MoS_{2-x}OH_y$ and Co–$MoS_{2-x}OH_y$ catalysts with the representative linear sweep voltammograms (LSVs) summarized in Supplementary Fig. 51a. The overpotentials for achieving geometric current density of 10 mA cm^{-2} are used for activity comparison. The value obtained for MoS_2 is 332 mV, which is consistent with previous studies. [2, 3]While after incorporating M-OH site, the overpotentials are significantly decreased to -165 and -203 mV for Ni-$MoS_{2-x}OH_y$ and Co-$MoS_{2-x}OH_y$, respectively. The decreased overpotential indicates that the metal hydroxide/ oxide sites (M-OH, M= Ni, Co) doping into MoS_2 backbone can improve the HER activity of MoS_2 catalysts. The corresponding Tafel plots (Supplementary Fig. 51b) show that Co and Ni doping decreases the Tafel slope from 162 to 90–101 mV dec^{-1}, demonstrating the transition of the RDS away from the Volmer discharge reaction ($H_2O + M + e^- \rightarrow M\text{-}H_{ads} + OH^-$). Therefore, we conclude that the metal hydroxide/ oxide sites induced activation of water, thus improving the HER performance of M-$MoS_{2-x}OH_y$, which is consistent with the previously observed role of 3d-TM cations in catalysing the HER on metal surfaces decorated by 3d-TM$(OH)_2$.

Supplementary Discussion 1

According to the experiment, we did extra calculation to achieve the Ru-OH formation energy and its influence on the Ru-S bond energy. As shown the Supplementary Figure 16, the formation energy of sulfur vacancy is decreased from 1.79 eV to 1.14 eV after forming the Ru-OH bond. Therefore, this site

is more prone to form SV after –OH substitution, thereby forming the site structure shown in Figure 3b, which is consistent with our EXAFS data.

Supplementary Discussion 2

We adopted ^{1}H SS-NMR spectrum to further prove the presence in O-H form.(Supplementary Figure 21) The ^{1}H SS-NMR spectrum of the Pd,Ru-$MoS_{2-x}OH_y$ catalyst shows a peak at 2.802 ppm, which could match a hydrogen atom bonded to an oxygen atom. Here, the peak at 5~7 ppm is attributed to the H_2O adsorption and the peak at 0.126~0.452 ppm belong to the standard sample peak. The counterparts Ru-MoS_2, MoS_2 and Pd-MoS_2 samples show no significant ^{1}H NMR peak at 2.802 ppm, which again identify that the oxygen-containing species in the Pd,Ru-$MoS_{2-x}OH_y$ catalyst is –OH.

Supplementary Discussion 3

Here, two different configurations were considered for the calculations, i.e., the Pd and Ru atoms are adjacent to each other (Supplementary Figure 43a-c) and Pd and Ru are not adjacent (Supplementary Figure 43a-c). It is noted that in the latter case, where Pd and Ru atoms do not form Pd-S-Ru bonds, the effect Pd on water adsorption energies on different sites can be neglected. Therefore, we chose Ru doping model instead of Ru, Pd co-doping model for calculation.

Supplementary Methods

Chemicals and materials. The sodium molybdate (Na_2MoO_4•$2H_2O$), thiourea (CH_4N_2S), palladium acetate ($Pd(OAc)_2$), $RuCl_3$ and 5wt% Nafion ionomer was obtained from Aldrich. Commercial state-of-the-art 20wt% Pt/C (Johnson Matthey Company, HiSPEC™ 3000) was used as the benchmark for comparison and was denoted as Pt/C-JM. Vulcan carbon powder XC-72 was purchased from Cabot Co. All the chemicals were used as delivered without further treatment. Highly purified argon (≥ 99.99%)

was supplied by Changchun Juyang Co Ltd. Ultrapure water (resistivity：ρ ≥ 18 MΩ cm-1) was used to prepare the solutions.

Physical Characterization

Scanning electron microscopy (SEM). SEM measurements were performed with an XL 30 ESEM FEG field emission scanning electron microscope.

X-ray photoelectron spectroscopy (XPS). XPS measurements were performed on a AXIS Ultra DLD (Kratos Company) using a monochromic Al X-ray source.

Powder X-ray diffraction (XRD). X-ray diffraction (XRD) measurements were performed with a PW-1700 diffractometer using a Cu Kα (λ=1.5405 Å) radiation source (Philips Co.).

Raman spectra (Raman). Raman spectra were collected on a J-Y T64000 Raman spectrometer with 514.5 nm wavelength incident laser light.

Transmission electron microscopy (TEM). A Philips TECNAI G2 electron microscope operating at 200 kV accelerating voltage was used for TEM, high resolution transmission electron microscopy (HRTEM), high-annular dark-field scanning transmission electron microscopy (STEM) and element mapping analysis.

Inductively Coupled Plasma-Atomic Emission Spectroscopy-Mass Spectrometry (ICP-AES-MS). The bulk compositions were evaluated by inductively coupled plasma optical emission spectrometer (X Series 2, Thermo Scientific USA). ll elemental analyses of catalyst samples were analyzed by ICP-AES-MS (Inductively Coupled Plasma-Atomic Emission Spectroscopy-Mass Spectrometry) on a Thermo Elemental IRIS Intrepid.

The high-angle annular dark-field scanning transmission electron microscopy (HAADF-STEM).The high-angle annular dark-field scanning transmission electron microscopy (HAADF-STEM) images

were obtained by using a Titan 80-300 scanning/transmission electron microscope operated at 300 KV, equipped with a probe spherical aberration corrector.

The detailed information about HER stability measurement of Pt catalyst as shown below:

All electrochemical measurements (cyclic voltammetry, galvanostatic polarization) were conducted in a conventional three-electrode cell under 30 ℃ using the 750E Bipotentiostat (CH Instruments). A reversible hydrogen electrode (RHE) served as the reference electrode, and a graphite plate served as the counter electrode, respectively. The catalyst ink was prepared by ultrasonically dispersing 1 mg Pt catalyst in a suspension containing 1 mL isopropanol, 10 μL Nafion (5 wt%) solution and 3.99 mL H_2O, then dropped the link on a glassy carbon rotating ring-disk electrode (RRDE) with a loading of 20 ug•cm^{-2} to form catalyst film coated electrode by drying in air with 500 rpm rotating. The cyclic voltammetry curves were investigated by continuous potential cycling in H_2-saturated 0.5 M H2SO4 solution between 0.1 V and -0.15 V with the scan rate at 0.1 V s^{-1}. And after 200 cycles, galvanostatic experiments were carried out directly after the linear polarization curves, whereby a constant current density was applied and the resulting potential was recorded over time.

The HER performance measurement of Pd,Ru-$MoS_{2-x}OH_y$ catalyst using the RDE method:

The electrochemical measurements were performed in a glass cell for rotating electrodes (PINE Research Instrumentation), with a reversible hydrogen electrode electrode as the reference electrode, a graphite plate served as the counter electrode and a 5 mm diameter glassy carbon as the working electrode. Cyclic voltammograms (CV) of Pd,Ru-$MoS_{2-x}OH_y$ was recorded between 0.03 to -0.15 V vs. RHE in H_2-saturated electrolytes at a scanning rate of 100 mV/s. Hydrogen evolution reaction (HER) polarization curves were obtained by RDE measurement in 0.1 M $HClO_4$ with saturated H_2, at a scanning rate of 10 mV/s and rotation speeds ranging from 400 rpm to 2500 rpm at r.t. (30℃).

Computational details.

The formation energy (E_f) for different S-vacancies were calculated to evaluate their structural stability, according to the formula $E_f = E_{SV} + E_S - E_{MoS2}$, where E_{SV} is the total energy for MoS_2 with S-vacancy, E_s is the energy for single S atom and E_{MoS2} is the total energy for MoS_2 without vacancy, respectively.

The adsorption energy (E_{ad}) of reaction moieties is calculated as the differences in total energy of the Ru-MoS_2 and the adsorbate and the pristine Ru-MoS_2 plus the adsorbate in the gas phase, according to equation (1):

$$E_{ad} = E_{adsorbate+Ru/MoS_2} - E_{Ru/MoS_2} - E_{adsorbate} \quad (1)$$

In order to probe the relationship between the electronic structure and the adsorption properties, the electron localization function (ELF) is employed. It is worth mentioning that ELF is more suitable than the electronic charge density in describing chemical bonds. The explicit formula of ELF is given by[4-6]

$$\mathrm{ELF} = \frac{1}{1 + \left(\frac{D(\vec{r})}{D_h(\vec{r})} \right)^2} \quad (2)$$

$$D(\vec{r}) = \frac{1}{2} \nabla_{\vec{r}} \nabla_{\vec{r}'} \rho(\vec{r}, \vec{r}') |_{\vec{r}=\vec{r}'} - \frac{1}{8} \frac{\left| \nabla n(\vec{r}) \right|^2}{n(\vec{r})} \quad (3)$$

$$D_h(\vec{r}) = \frac{3}{10} (3\pi^2)^{2/3} n(\vec{r})^{5/3} \quad (4)$$

where ρ is the first-order reduced spin-integrated density matrix. $D(\vec{r})$ is the von Weizsäcker kinetic-energy functional and $D_h(\vec{r})$ is the kinetic-energy density of a uniform electron gas with a spin density equal to the local value of $n(\vec{r})$.

The projected density of states (pDOS) were performed to study the bonding and electronic structure between -OH and H_2O. Here, we calculated the PDOS of O and H using the data related to the H and O involved in hydrogen bonding instead of all the H and O atoms.

Supplementary References

1. Yu, L., Xia, B.Y., Wang, X. & Lou, X.W. General formation of M-MoS_3 (M = Co, Ni) hollow structures with enhanced electrocatalytic activity for hydrogenevolution. *Adv. Mater.* **28**, 92-97 (2016).
2. Li, G. et al. All The Catalytic Active Sites of MoS_2 for Hydrogen Evolution. *J. Am. Chem. Soc.* 138, 16632-16638 (2016).
3. Yin, Y. et al. Contributions of phase, sulfur vacancies, and edges to the hydrogen evolution reaction catalytic activity of porous molybdenumdisulfide nanosheets. *J. Am. Chem. Soc.* **138**, 7965-7972 (2016).
4. Tsai, C. et al. Electrochemical generation of sulfur vacancies in the basal plane of MoS_2 for hydrogen evolution. *Nat. Commun.* **8**, 15113 (2017).
5. He, R. et al. Molybdenum Disulfide-Black Phosphorus Hybrid Nanosheets as a Superior Catalyst for Electrochemical Hydrogen Evolution. *Nano letters* **17**, 4311-4316 (2017).
6. Konkena, B., Masa, J., Xia, W., Muhler, M. & Schuhmann, W. MoSSe@reduced graphene oxide nanocomposite heterostructures as efficient and stable electrocatalysts for the hydrogen evolution reaction. *Nano Energy* **29**, 46-53 (2016).
7. Deng, J. et al. Triggering the electrocatalytic hydrogen evolution activity of the inert two-dimensional MoS_2 surface via single-atom metal doping. *Energy Environ. Sci.* **8**, 1594-1601 (2015).
8. Li, H. et al. Corrigendum: Activating and optimizing MoS_2 basal planes for hydrogen evolution through the formation of strained sulphur vacancies. *Nat. Mater.* **15**, 364 (2016).
9. Kiriya, D. et al. General Thermal Texturization Process of MoS_2 for Efficient Electrocatalytic Hydrogen Evolution Reaction. *Nano letters* **16**, 4047-4053 (2016).
10. Deng, J. et al. High-performance hydrogen evolution electrocatalysis by layer-controlled MoS_2 nanosheets. *RSC Advances* **4**, 34733 (2014).
11. Geng, X. et al. Pure and stable metallic phase molybdenum disulfide nanosheets for hydrogen evolution reaction. *Nat. Commun.* **7**, 10672 (2016).
12. Gao, M.R. et al. An efficient molybdenum disulfide/cobalt diselenide hybrid catalyst for electrochemical hydrogen generation. *Nat. Commun.* **6**, 5982 (2015).
13. Tran, P.D. et al. Coordination polymer structure and revisited hydrogen evolution catalytic mechanism for amorphous molybdenum sulfide. *Nat. Mater.* **15**, 640-646 (2016).
14. Shi, Y. et al. Energy Level Engineering of MoS_2 by Transition-Metal Doping for Accelerating Hydrogen Evolution Reaction. *J. Am. Chem. Soc.* **139**, 15479-15485 (2017).
15. Luo, Z. et al. Chemically activating MoS_2 via spontaneous atomic palladium interfacial doping towards efficient hydrogen evolution. *Nat. Commun. **9**, 2120 (2018).*.
16. Zhang, B. et al. Interface engineering: The $Ni(OH)_2/MoS_2$ heterostructure for highly efficient alkaline hydrogen evolution. *Nano Energy **37**, 74-80 (2017).*
17. Zhang, J. et al. Interface Engineering of MoS_2/Ni_3S_2 Heterostructures for Highly Enhanced Electrochemical Overall-Water-Splitting Activity. *Angew. Chem. Int. Ed.* **55**, 6702-6707 (2016).
18. Kamran, S., Chen, K.Y. & Chen, L. Ab initio examination of ductility features of fcc metals. *Phy. Rev. B* **79**, 11 (2009).
19. De Santis, L. & Resta, R. Electron localization at metal surfaces. *Surf. Sci.* **450**, 126-132 (2000).

20. Krokhin, A.A., Makarov, N.M., Yampolskii, V.A. & Yurkevich, I.V. Problem of Localization and Scattering of the Conduction Electrons at the Rough Metal-Surface. *Physica B* **165**, 855-856 (1990).
